# Agrimoniin Alleviates Ferroptosis in Cold‐Stored DCD Liver Grafts Through Activation of the Nrf‐2 Pathway

**DOI:** 10.1111/cpr.70164

**Published:** 2026-01-20

**Authors:** Enqiang Chang, Xiaoting Liao, Guanghua Tao, Bijun Luo, Sheng He, Linghui Pan

**Affiliations:** ^1^ Department of Anesthesiology Guangxi Medical University Cancer Hospital, Guangxi Clinical Research Center for Anesthesiology Nanning China; ^2^ Division of Anaesthetics, Pain Medicine and Intensive Care, Department of Surgery and Cancer, Faculty of Medicine Imperial College London, Chelsea and Westminster Hospital London UK; ^3^ Department of Anesthesiology Xichang People's Hospital Xichang China; ^4^ Department of Anesthesiology The Maternal and Child Health Care Hospital of Guangxi Zhuang Autonomous Region Nanning China; ^5^ Department of Anesthesiology, the First Affiliated Hospital, Hengyang Medical School University of South China Hengyang China

**Keywords:** agrimoniin, ferroptosis, liver transplantation, Nrf‐2 pathway

## Abstract

Liver grafts from donation‐after‐cardiac‐death (DCD) are vulnerable to ischemia–reperfusion injury, which compromises graft function after transplantation. Agrimoniin has been shown to possess antioxidant and anti‐inflammatory properties, making it a potential therapeutic agent for organ preservation. This study investigated whether supplementing agrimoniin to the University of Wisconsin (UW) cold storage solution protected liver grafts from DCD rats or cold preserved human liver cell lines (QSG‐7701 and HepG2). Agrimoniin supplementation significantly reduced oxidative damage, alleviated ferroptosis, and mitigated liver injury by activating the Nrf‐2 pathway, both in vivo and in vitro. These findings suggest that ferroptosis is a mediator in DCD liver injury, and agrimoniin, through its activation of the Nrf‐2 pathway, may be an effective therapeutic agent for enhancing liver graft preservation and improving outcomes in DCD liver transplantation.

## Graphical abstract

Supplementing the liver preservation solution with agrimoniin protects donation after cardiac death (DCD) grafts by activating the Nrf‐2 pathway. This reduces ferroptosis, oxidative stress, and inflammation during cold storage, significantly improving graft viability.

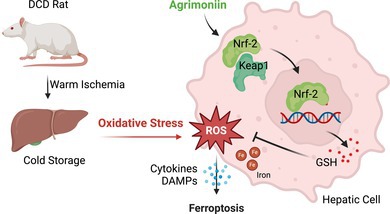

**Abbreviations**
ACSL4: long‐chain‐fatty‐acid—CoA ligase 4
ALT: alanine aminotransferase
AST: aminotransferase
COX‐2: cyclooxygenase‐2
DCD: donation after cardiac death
DGF: delayed graft function
GAPDH: glyceraldehyde 3‐phosphate dehydrogenase
GPX4: Glutathione Peroxidase 4
GSH: glutathione
GSSG: glutathione disulfide
H&E: haematoxylin and eosin
Keap1: Kelch‐like ECH‐associated protein 1
NQO1: NAD(P)H quinone oxidoreductase
Nrf‐2: nuclear factor erythroid 2‐related factor 2
PGD: primary graft dysfunction
ROS: reactive oxygen species
SOD: superoxide dismutase
TNF‐α: tumour necrosis factor alpha
UW: University of Wisconsin

## 1 Introduction

Liver transplantation using donors after cardiac death (DCD) offers additional opportunities for patients awaiting liver transplants [1, 2]. DCD donors are characterised as death declared based on cardiopulmonary criteria rather than the cessation of brain function [3]. The use of DCD donors effectively addresses the organ availability gap for transplantation and enlarges the pool of potential organ donors [4]. However, DCD livers may pose a higher risk of primary nonfunction, leading to poor outcomes for recipients due to severe ischemia–reperfusion (IR) injury [5]. Additionally, DCD livers carry an increased risk of anastomotic and intrahepatic biliary strictures [6]. Therefore, preserving excised DCD livers in a cold storage solution at low temperatures is crucial in DCD liver transplantation.

While cold preservation mitigates damage to the liver, prolonged storage in cold conditions may have adverse effects. Hence, in DCD liver transplantation, monitoring liver physiological parameters and implementing necessary measures to minimise potential damage induced by cold ischemia is crucial to transplant success [7]. Research aimed at improving organ preservation solutions primarily focuses on supplementing protective drugs, such as proteasome inhibitors [8] and anti‐ischemic drugs [9], into the storage solution and has shown promise in experimental settings. Agrimoniin, a polyphenolic compound present in various plants, particularly in the agrimonia genus, is known for its antioxidant and anti‐inflammatory properties [10]. It is reported that agrimoniin can neutralise free radicals and protect cells from oxidative damage and an inflammatory environment [11].

Hence, we propose the hypothesis that the addition of agrimoniin to the storage solution could enhance the quality of DCD livers. This study employed a DCD rat model to investigate the antioxidant and anti‐inflammatory effects of agrimoniin on prolonged cold‐stored DCD livers. We also aimed to explore the underlying molecular mechanisms, including the Nuclear factor erythroid 2‐related factor 2 (Nrf‐2) antioxidant pathway and ferroptosis pathway.

## 2 Material and Methods

### 2.1 Animals and DCD Model

Specific‐pathogen‐free (SPF) male Sprague‐Dawle (SD) rats weighing 230–330 g were purchased from Vital River Laboratory Animal Technology Co. Ltd. (Beijing, China) and housed in temperature‐ and humidity‐controlled cages in a specific pathogen‐free facility at the Laboratory Animal Platform of Zhengzhou University Academy of Medical Sciences. All experiments were conducted following the ARRIVE guidelines and in accordance with the principles of laboratory animal care provided by the National Institutes of Health [12], and the use of laboratory animals was approved and monitored by the Laboratory Animal Care and Ethics Committee of Zhengzhou University (No. ZZU‐LAC20210931[06]).

The Rat DCD model was established according to a previous study [13]. Briefly, the rats were anaesthetised with an intraperitoneal injection of pentobarbital sodium (5 mg/100 g, Sigma‐Aldrich, St. Louis, United States) and placed on a heating pad to maintain the body temperature. To achieve anticoagulation, 250 U Heparin with saline was injected through the penile vein. Five minutes after heparinization, cardiac arrest was induced by external compression of the heart. After a warm ischemia period of 30 min, the entire liver was flushed with the cold University of Wisconsin (UW) solution (Bridge to Life Ltd., New York, USA) supplemented with or without 25 or 100 μM agrimoniin (Sigma‐Aldrich, St. Louis, United States). Then, the livers were excised and cold‐preserved in the UW solution supplemented with or without agrimoniin at 4°C for 24 h. A liver graft from a living donor, serving as the naive control, was excised immediately after anaesthesia.

### 2.2 Administration of Agrimoniin and Nrf‐2 siRNA

The dissolved (200 μg in 10 mL of PBS) Nrf‐2 siRNA or scrambled siRNA (Qiagen, Crawley, West Sussex, UK) was swiftly injected via a tail vein within 30 s while the rats were under anaesthesia. The rats were allowed to recover for 24 h before cardiac death surgery. Agrimoniin at a dose of 20 mg/kg or 100 mg/kg was administered intraperitoneally 24 h prior to graft extraction.

### 2.3 In Vitro Cell Culture and Treatments

Human normal liver cells (QSG 7701 cell line) and human hepatocellular carcinoma cells (HepG2 cell line) were purchased from Beyotime company (Hubei, China). The cells were cultured in RPMI 1640 or DMEM cell culture media with 10% FBS (Gibco) and 100 U/mL penicillin–streptomycin (Gibco) in an incubator at 37°C. The cells were cold preserved in the UW preservation solution saturated with 100 μM agrimoniin and/or the dissolved (200 μg in 10 mL of PBS) Nrf‐2 siRNA or scrambled siRNA at 4°C for 24 h.

### 2.4 Haematoxylin and Eosin (H&E) Staining

Five micrometres of paraffin‐embedded liver sections were deparaffined by immersing slides in xylene. Gradually rehydrate sections and then immerse slides in Haematoxylin solution and then Eosin Y solution. All sections were visualised under a light microscope at ×20 magnification (Nikon, Tokyo, Japan), and 10 pictures were captured randomly for each section. Damage to the livers was scored from 0 to 4 degrees system: Grade 0, normal liver appearance; Grade 1, mild liver structure damage and the cell death rate range from 0%–25%; Grade 2, moderate liver structure damage and the cell death rate range from 25%–50%; Grade 3, severe destruction of the liver architecture and the cell death rate range from 50%–75%; Grade 4, the liver architecture is totally destroyed and the cell death rate range from 50%–75%.

### 2.5 Iron Staining

The slides underwent a 3‐min incubation in the active iron stain solution (Abcam, Cambridge, UK), followed by 5 min of exposure to a nuclear fast red solution. Afterwards, the sections underwent rinsing and dehydration and were ultimately mounted in synthetic resin. All sections were examined using a widefield microscope at 20× magnifications (Nikon, Tokyo, Japan).

### 2.6 Oxidative Stress Assay

The slides were incubated with primary antibody (1:100) overnight at 4°C and biotinylated secondary antibody for 30 min at room temperature. DAB mixture was used for colour development. Nuclear were stained with haematoxylin. All reagents were from OxyIHC Oxidative Stress Detection Kit (EMD Millipore, S7450, Poole, UK). Finally, the slides were dehydrated, immersed in xylene and mounted in synthetic resin.

### 2.7 Glutathione (GSH) and Glutathione Disulfide (GSSG) Detection

A glutathione assay kit (Sigma‐Aldrich, St. Louis, United States) was used to evaluate GSH and GSSG. Homogenised and centrifuged the tissue or cell lysate to extract total protein. Measured the protein concentration in the samples using a Bradford assay. After creating a standard curve, incubated the samples and standards with the reaction mixture for 1 h, and then measured the absorbance of the reaction mixture using a spectrophotometer.

### 2.8 TUNEL Assay

The slides were incubated with DNA labelling solution (Abcam, Cambridge, UK) for 1 h in dark humidified 37°C incubator, followed by 30 min antibody solution and 30 min staining buffer incubation in the dark at room temperature. The nuclear were stained by 4′,6‐diamidino‐2‐phenylindole (DAPI) and the slides were covered with mounting solution and coverslip for detection.

### 2.9 Immunofluorescence

The tissues or cells underwent permeabilization with 0.1% Triton. To block non‐specific binding, tissue sections were treated with 5% donkey serum (Sigma‐Aldrich, St. Louis, United States) in PBS containing 0.3% Triton X‐100 (PBS‐T) for 30 min at room temperature. Then, the primary antibodies, including acyl‐CoA synthetase long chain family member 4 (ACSL4), Glutathione Peroxidase 4 (GPX4), Nuclear factor erythroid 2‐related factor 2 (Nrf‐2), NAD(P)H quinone oxidoreductase (NQO1), and Kelch‐like ECH‐associated protein 1 (Keap1) (Abcam, Cambridge, UK), were diluted in 1:100 with PBS‐T, were applied and left to incubate the tissue sections overnight at 4°C. Subsequently, the tissue sections were washed three times with PBS‐T. A fluorochrome‐conjugated secondary antibody (Abcam, Cambridge, UK) was incubated at room temperature for 1 h, followed by three washes with PBS‐T. Finally, the samples were treated with DAPI, mounting solution (Vector Laboratories, Burlingame, USA) and prepared for examination.

### 2.10 Western Blot

Fresh liver tissues were homogenised in liquid nitrogen and centrifuged at 10,000 g for 20 min at 4°C to remove debris. After being heated, denatured and loaded onto a NuPAGE 4 to 12% Bis‐Tris gel for electrophoresis, the protein extracts (40 g/sample) were transferred to a polyvinylidene difluoride membrane and blocked with 5% BSA in TBST for an hour at room temperature. Membranes were probed with 1:1000 primary antibodies GPX4 (Abcam), ACSL4 (Abcam), CHOP (Abcam), and GRP78 (Abcam) primary antibodies in TBS‐T overnight at 4°C, followed by HRP‐conjugated secondary antibody for 1 h (Cell Signalling, Technology). The loading control was the constitutively expressed protein, Glyceraldehyde 3‐phosphate dehydrogenase (GAPDH, Cell Signalling, Technology). The blots were visualised using improved chemiluminescence (Santa Cruz, Dallas, USA). The expression of protein bands compared to non‐cold‐stored control samples was normalised to GAPDH expression.

### 2.11 Cell Viability

Five milligrams per millilitre of MTT reagent (Beyotime, Hubei, China) was added to each well and incubated the cells at 37°C for 3 h in the dark. After dissolved the formazan crystals with DMSO, the medium absorbance was measured using a spectrophotometer.

### 2.12 Determination of Liver Enzymes and Cytokines and DAMPs Levels

Liver enzymes aspartate aminotransferase (AST; ab263883) and alanine aminotransferase (ALT; ab234579), as well as pro‐inflammatory cytokines Tumour necrosis factor alpha (TNF‐α) (ab100785), IL‐18 (ab213909), and IL‐1β (ab100768) were measured with rat or human recombinant ELISA kits (Abcam, Cambridge, UK). Human (EEL047) and mouse (EEL102) ELISA kits (Invitrogen, USA) were used to measure tissue and extracellular Human High Mobility Group Protein B1 (HMGB1) level. Histone level was detected by PathScan Total Histone H3 Sandwich ELISA Kit (CST; #7253).

### 2.13 Statistical Analysis

All numerical data were expressed as mean ± standard deviation (SD) and presented with a bar and scatter plot. Data were analysed using the analysis of variance (ANOVA) with post hoc Bonferroni test for comparisons where appropriate (GraphPad Prism 5.0, GraphPad Software). A *p*‐value < 0.05 was considered to be of statistical significance.

## 3 Results

### 3.1 Agrimoniin Injection and Supplementation Reduced DCD Liver Injury and Apoptosis

**FIGURE 1:**
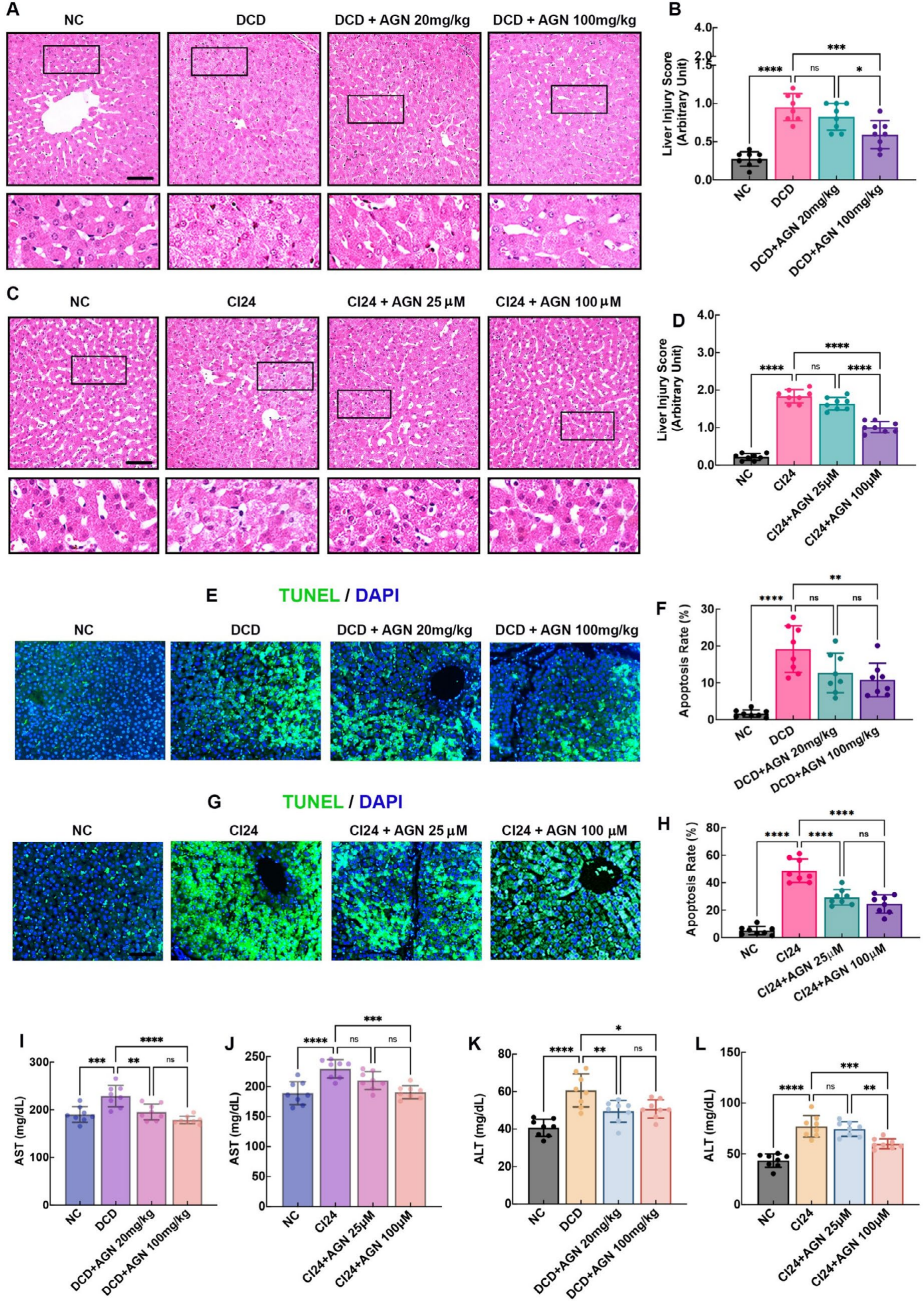
Agrimoniin injection and supplementation reduced DCD liver injury. Intraperitoneally injection of agrimoniin 20 mg/kg or 100 mg/kg 24 h before DCD surgery, liver grafts were harvested 30 min after cardiac death. (A) H&E staining was conducted and (B) liver injury score was assessed to evaluate liver graft injury. The DCD liver grafts were harvested 30 min after cardiac death and then were cold‐preserved in the UW solution supplemented with 25 or 100 μM agrimoniin for 24 h. (C) H&E staining was conducted and (D) liver injury score was assessed to evaluate liver graft injury. (E and F) TUNEL (green) and staining were conducted in the liver upon 24 h of cold preservation and (F and H) the apoptosis rate were evaluated. The concentrations of enzymes (I and K) ALT and (J and L) AST were evaluated. Nuclei were counterstained with DAPI (blue). Data were analysed using ANOVA with post hoc Bonferroni test and are presented as bars with scatter plots, mean ± SD. *n* = 8. Scale bar = 200 μm. *p < 0.05, **p < 0.01, ***p < 0.001, ****p < 0.0001.

A morphological evaluation of the liver grafts from DCD rats with agrimoniin intraperitoneally pretreatment (Figure 1A). The results showed that cardiac death and warm ischemia (DCD) significantly damaged liver architecture and increased liver cell death, as well as increased liver injury score (*p* < 0.0001, Figure 1B). Intraperitoneally injected agrimoniin 100 mg/kg, but not 20 mg/kg 24 h before cardiac death significantly reduced liver cell death and decreased liver injury score (*p* < 0.001, Figure 1B). In addition, DCD livers cold preserved for 24 h (CI24) further increased liver architecture damage (Figure 1C) and increased liver injury score (*p* < 0.0001, Figure 1D). A remarkable improvement in liver morphology and a decreased liver injury score (*p* < 0.0001, Figure 1D) were observed when the cold storage solution was supplemented with 100 μM agrimoniin.

Liver cell death was assessed through TUNEL assay (Figure 1E,G). DCD livers exhibited significant rise in percentage of TUNEL+ apoptotic cells (*p* < 0.0001) while injection of 100 mg/kg agrimoniin significantly reduced cell death (*p* < 0.01, Figure 1F). CI24 further aggravated cell death in DCD liver graft (*p* < 0.0001, Figure 1H). Notably, Cold storage solution supplemented either with 25 or 100 μM agrimoniin significantly decreased the percentage of apoptotic cells (*p* < 0.0001, Figure 1H). Moreover, both DCD and cold storage (CI24) caused a significant increase in AST (Figure 1I,J) and ALT (Figure 1K,L) levels. Intraperitoneally injected agrimoniin 20 mg/kg (*p* < 0.01, respectively) or 100 mg/kg (*p* < 0.05, *p* < 0.0001, respectively) significantly decreased AST and ALT levels (Figure 1I,K). Meanwhile, 100 μM agrimoniin supplementation in the cold storage solution also significantly reduced AST and ALT levels (*p* < 0.001, respectively; Figure 1J,L).

### 3.2 Agrimoniin Injection and Supplementation Reduced DCD Liver Ferroptosis

**FIGURE 2:**
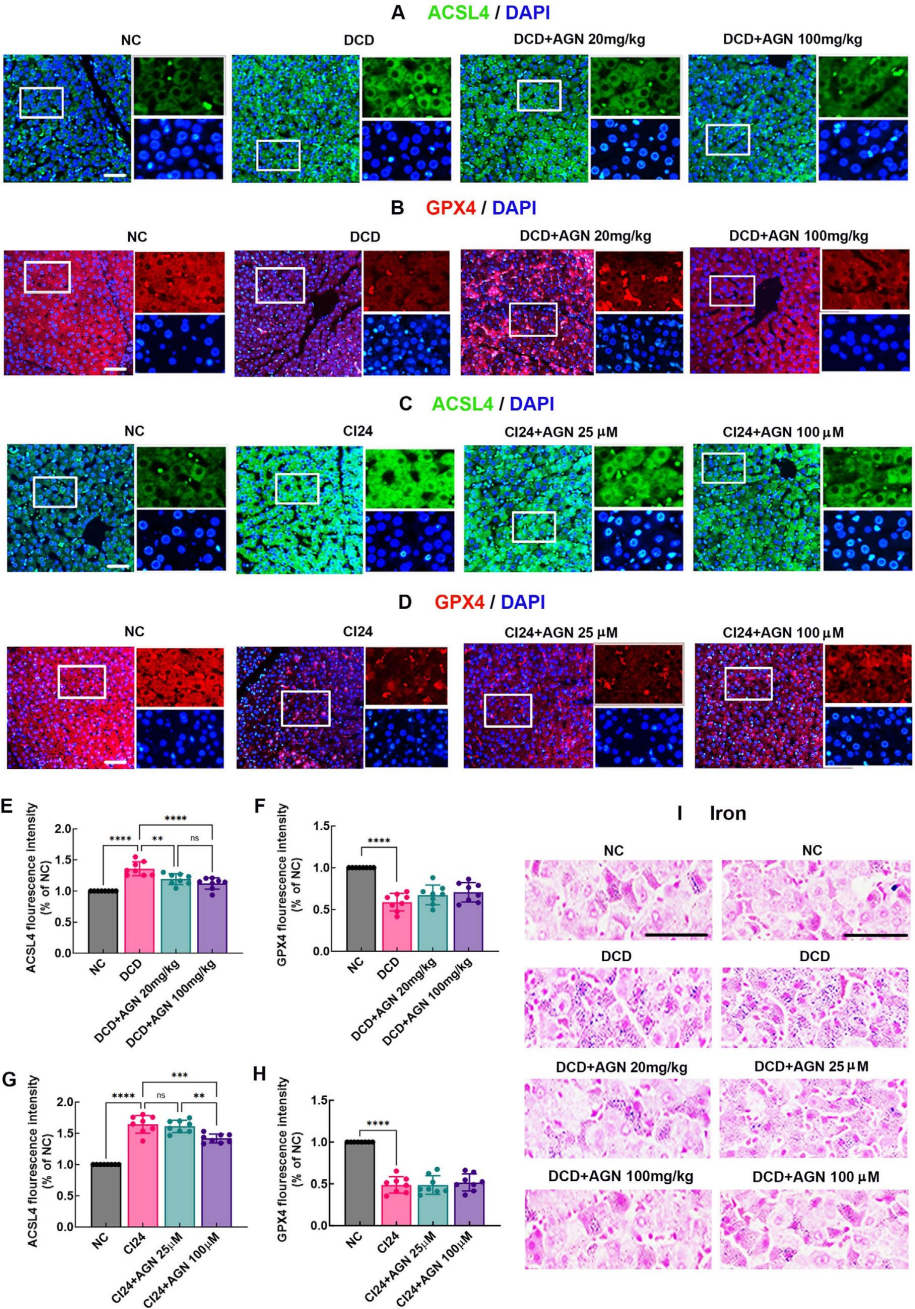
Agrimoniin injection and supplementation reduced DCD liver ferroptosis. Intraperitoneally injection of agrimoniin 20 mg/kg or 100 mg/kg 24 h before DCD surgery, liver grafts were harvested 30 min after cardiac death. (A) ACSL4 (green) staining in the liver. (B) GPX4 (red) staining in the liver. (E) Average ACSL4 fluorescence intensity. (F) Average GPX4 fluorescence intensity. The DCD liver grafts were harvested 30 min after cardiac death and then were cold‐preserved in the UW solution supplemented with 25 or 100 μM agrimoniin for 24 h. (C) ACSL4 (green) staining in the liver. (D) GPX4 (red) staining in the liver. (G) Average ACSL4 fluorescence intensity. (H) Average GPX4 fluorescence intensity. (I) Iron staining of liver grafts upon 24 h cold storage. Nuclei were counterstained with DAPI (blue). Data were analysed using ANOVA with post hoc Bonferroni test and are presented as bars with scatter plots, mean ± SD. *n* = 8. Scale bar = 200 μm. **p < 0.01, ***p < 0.001, ****p < 0.0001.

The expressions of GPX4 and ACSL4, specific biomarkers of ferroptosis, were evaluated to determine the occurrence of ferroptosis (Figure 2A–H). The results showed that cardiac death and warm ischemia (DCD) significantly increased ACSL4 expression (*p* < 0.0001, Figure 2A,E) and decreased GPX4 expression (*p* < 0.0001, Figure 2B,F), indicating ferroptosis. Intraperitoneally injected agrimoniin 20 mg/kg (*p* < 0.01, Figure 2A,B,E,F) or 100 mg/kg (*p* < 0.0001, Figure 2A,B,E,F), or supplementing 100 μM (*p* < 0.001, Figure 2C,D,G,H) agrimoniin in the cold storage solution significantly reduced ACSL4, but not GPX4, expression, demonstrating the anti‐ferroptosis effect of agrimoniin. Meanwhile, DCD also increased iron deposition in the DCD liver grafts and cold preserved livers (CI24) and intraperitoneally injected agrimoniin or agrimoniim supplementation may reduce the iron deposition (Figure 2I).

### 3.3 Agrimoniin Supplementation Activated the Nrf‐2 Pathway in DCD Livers

**FIGURE 3:**
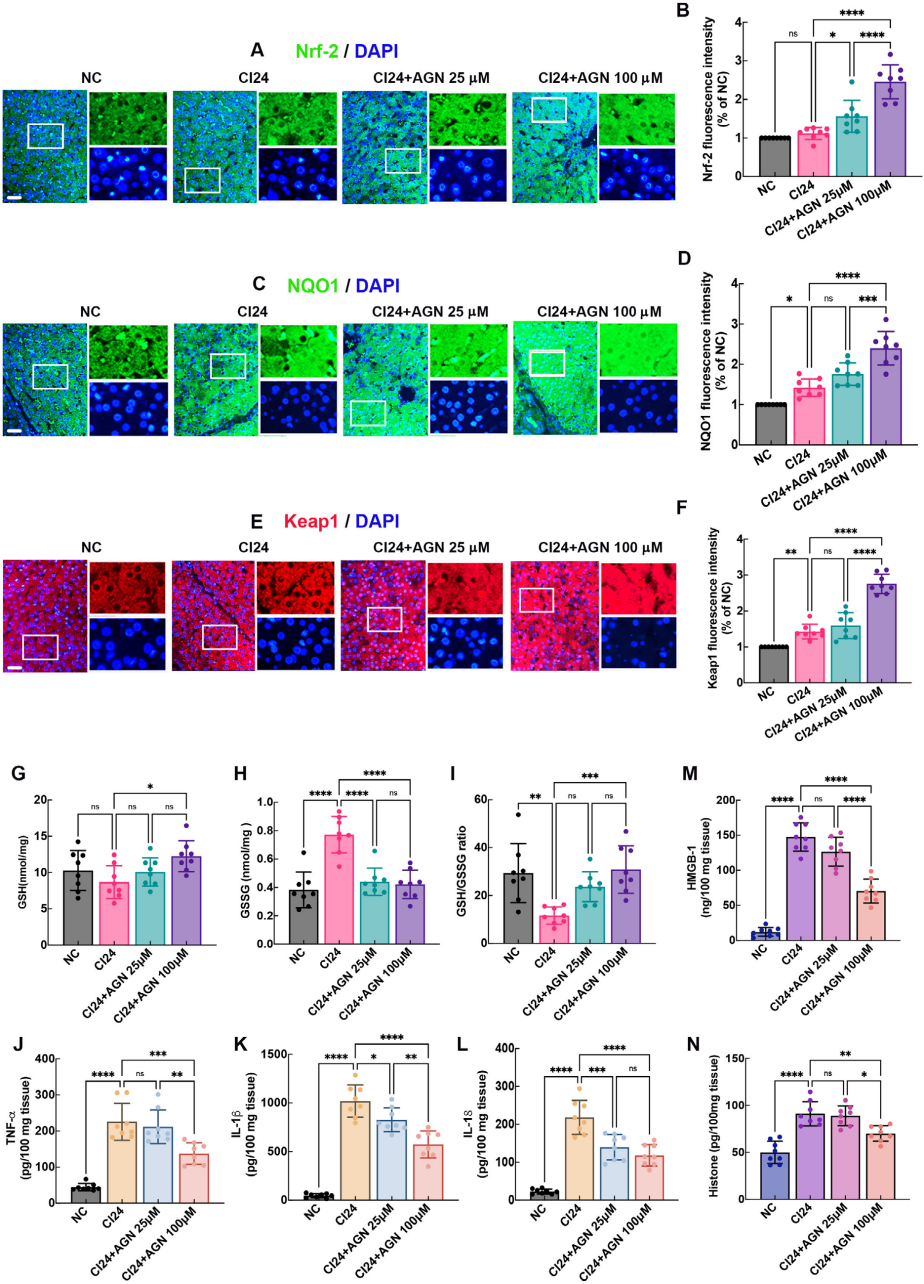
Agrimoniin supplementation activated the Nrf‐2 pathway in DCD livers. The DCD liver grafts were harvested 30 min after cardiac death and then were cold‐preserved in the UW solution supplemented with 25 or 100 μM agrimoniin for 24 h. (A) Nrf‐2 (green) staining of the liver. (B) Average Nrf‐2 fluorescence intensity. (C) NQO1 (green) staining of the liver. (D) Average NQO1 fluorescence intensity. (E) Keap1 (red) staining of the liver. (F) Average Keap1 fluorescence intensity. Nuclei were counterstained with DAPI (blue). The level of (G) GSH, (H) GSSH, (I) GSH/GSSG ratio, (M) HMGB1, (J) TNF‐α, (K) IL‐1β, (L) IL‐18, (G) and (N) Histone were evaluated by ELISA. Data were analysed using ANOVA with post hoc Bonferroni test and are presented as bars with scatter plots, mean ± SD. *n* = 8. Scale bar = 200 μm. *p < 0.05, **p < 0.01, ***p < 0.001, ****p < 0.0001.

The activation of Nrf‐2 and its downstream regulated enzymes, NQO1 and Keap1, mediate the antioxidative and anti‐inflammation activities in ischemia injury. Therefore, the protective effects of agrimoniin were evaluated by assessing Nrf‐2 pathway activation (Figure 3). The results showed that cold storage for 24 h did not increase Nrf‐2 expression (Figure 3A,B) but increased the expression of NQO1 (*p* < 0.05, Figure 3C,D) and Keap1 (*p* < 0.01, Figure 3E,F). Supplementation of 25 μM agrimoniin into the cold storage solution increased Nrf‐2 expression (*p* < 0.05, Figure 3B), while 100 μM agrimoniin significantly activated the Nrf‐2 pathway by increasing the expression of Nrf‐2 (Figure 3B), NQO1 (Figure 3D), and Keap1 (Figure 3F) (*p* < 0.0001, respectively). GSH as the most abundant antioxidant in cells, is modulated by Nrf‐2 pathway. The ratio of reduced GSH to oxidised GSH (GSSG) is an indicator of oxidative stress. CI24 significantly increased hepatic GSSG level and decreased GSH/GSSG ratio (*p* < 0.0001 and *p* < 0.01, respectively, Figure 3H,J), indicating rise in oxidative stress. Supplementation of the cold storage solution with 100 μM agrimoniin effectively restored GSH and GSSG level, and significantly improved the GSH/GSSG ratio (*p* < 0.05, p < 0.0001, *p* < 0.001 respectively, Figure 3G–I). Additionally, CI24 livers exhibited elevated levels of pro‐inflammatory cytokines TNF‐α (*p* < 0.0001, Figure 3J), IL‐1β (p < 0.0001, Figure 3K) and IL‐18 (p < 0.0001, Figure 3L) and DAMPs HMGB‐1 (*p* < 0.0001, Figure 3M) and histone (*p* < 0.0001, Figure 3N). Treatment with 100 μM agrimoniin significantly attenuated these inflammatory markers (*p* < 0.1 to *p* < 0.0001, Figure 3M,N).

### 3.4 Agrimoniin Supplementation Reduced Oxidative Stress Through Nrf‐2pathway in DCD Livers

**FIGURE 4:**
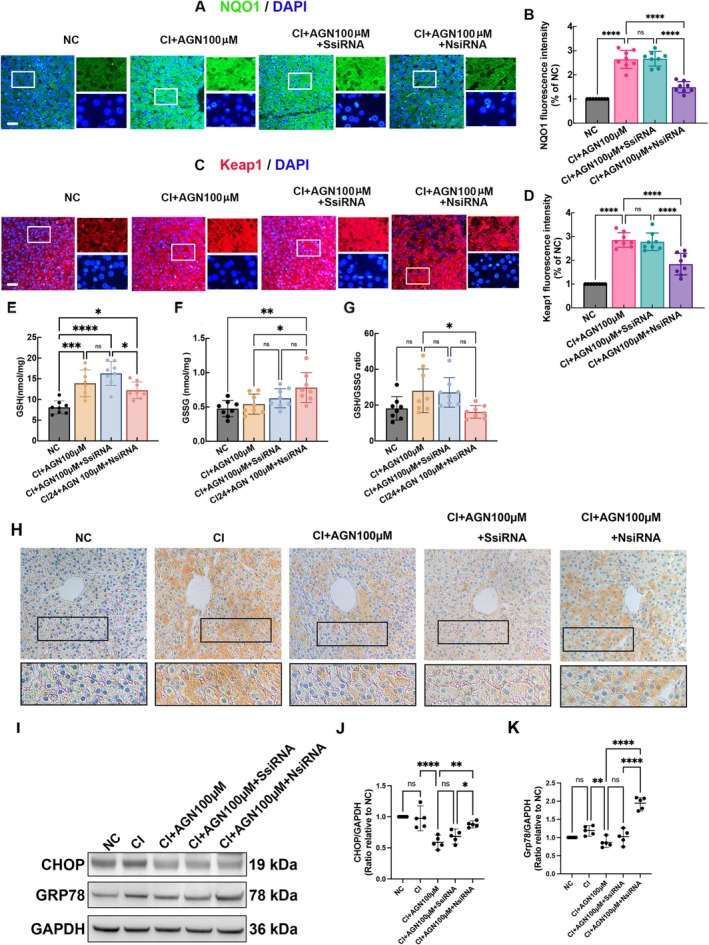
Agrimoniin supplementation reduced oxidative stress through Nrf‐2 pathway in DCD livers. The DCD liver grafts were harvested 30 min after cardiac death and then were cold‐preserved in the UW solution supplemented with 25 or 100 μM agrimoniin for 24 h. (A) NQO1 (green) staining of the liver. (B) NQO1 fluorescence intensity. (C) Keap1 (red) staining of the liver. (D) Keap fluorescence intensity. The concentration of (E) GSH, (F) GSSG in liver tissues were assessed by ELISA and the GSH/GSSG ratio were calculated (H) Tissue oxidative stress state was evaluated by OxyIHC oxidative stress detecting assay. (I) Endoplasmic reticulum stress was evaluated by detecting CHOP and GRP78 using Western Blot. Analysis shows the expression of (J) CHOP and (K) GRP78. Data were analysed using ANOVA with post hoc Bonferroni test and are presented as bars with scatter plots, mean ± SD. *n* = 8 or 5. *p < 0.05, **p < 0.01, ***p < 0.001, ****p < 0.0001.

Then, to confirm that the activation of the Nrf‐2 pathway mediates the protective effects of agrimoniin on cold‐preserved DCD liver grafts, Nrf‐2 siRNA (NsiRNA) was administrated to rats before liver extraction. The results showed that Nrf‐2 suppression significantly reduced the activation of the Nrf‐2 pathway induced by agrimoniin supplementation, as evidenced by the reduced expression of NQO1, and Keap1 (*p* < 0.0001, respectively; Figure 4A–D). Nrf‐2 knockdown abolished the antioxidative effects of agrimoniin. NsiRNA significantly decreased GSH level (*p* < 0.05, Figure 4E) and increased GSSG level (*p* < 0.05, Figure 4F), leading to a reduced GSH/GSSG ratio compared to the agrimoniin treated group (*p* < 0.05, Figure 4G). Correspondingly, the oxidative assay demonstrated that agrimoniin markedly alleviated oxidative stress in CI24 liver graft and this protective effect was diminished by NsiRNA (Figure 4H), suggesting that the anti‐oxidative effect of agrimoiin is largely dependent on Nrf‐2 pathway. Moreover, endoplasmic reticulum (ER) stress markers CHOP and GRP78 were evaluated. CI‐induced ER stress was significantly attenuated by agrimoniin treatment, as shown by reduced CHOP and GRP78 expression (*p* < 0.0001 and *p* < 0.01, respectively, Figure 4J,K), while this inhibitory effect on ER stress was abolished by Nrf‐2 knockdown.

### 3.5 Nrf‐2 Suppression Attenuated Agrimoniin‐Mediated Ferroptosis Reduction in DCD Livers

**FIGURE 5:**
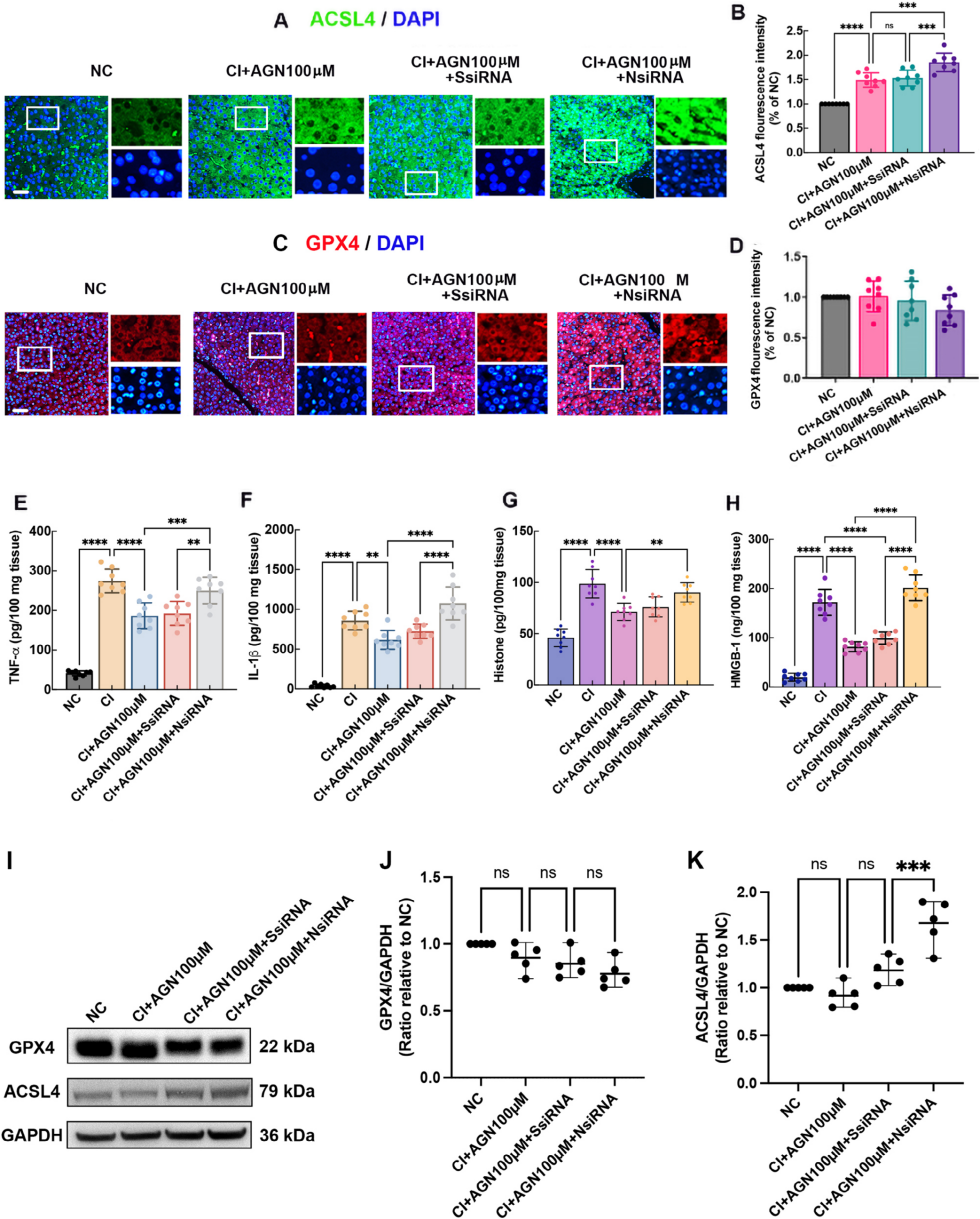
Nrf‐2 suppression attenuated agrimoniin‐mediated ferroptosis reduction in DCD livers. The DCD liver grafts were harvested 30 min after cardiac death and then were cold‐preserved in the UW solution supplemented with 25 or 100 μM agrimoniin for 24 h. Nrf‐2 siRNA (NsiRNA) or scrambled siRNA (SsiRNA) was given to donor rats before agrimoniin injection. (A) ACSL4 (green) staining of the liver. (B) Average ACSL4 fluorescence intensity. (C) GPX4 (red) staining of the liver. (D) Average GPX4 fluorescence intensity. The concentration of (E) TNF‐α, (F) IL‐1β, (G) Histone, and (H) HMGB‐1 in liver tissues were assessed by ELISA. (I) Ferroptosis was evaluated by detecting GPX4 and ACSL4 using Western Blot. Analysis shows the expression of (J) GPX4 and (K) ACSL4. Nuclei were counterstained with DAPI (blue). Data were analysed using ANOVA with post hoc Bonferroni test and are presented as bars with scatter plots, mean ± SD. *n* = 8 or 5. Scale bar = 200 μm. *p < 0.05, **p < 0.01, ***p < 0.001, ****p < 0.0001.

Nrf‐2 suppression further enhanced ferroptosis in cold‐preserved liver grafts by increasing ACSL4 expression when compared to liver grafts cold‐preserved in the agrimoniin‐supplemented storage solution (*p* < 0.0001, Figure 5A,B). GPX4 expression, however, did not show a significant change (*p* > 0.05, Figure 5C,D). Moreover, Nrf‐2 suppression significantly reversed the effects of agrimoniin on reducing pro‐inflammatory and DAMPs, as evidenced by increasing the levels of TNF‐α (*p* < 0.001, Figure 5E), IL‐1β (*p* < 0.0001, Figure 5F), Histone (*p* < 0.01, Figure 5G), and HMGB‐1 (*p* < 0.0001, Figure 5H). Western Blot results confirmed the significant increase in ACSL4 protein expression following Nrf‐2 knockdown (*p* < 0.001, Figure 5I,K), suggesting that the ferroptosis‐inhibitory effect of agrimoniin is mediated through Nrf‐2 pathway.

### 3.6 Agrimoniin Supplementation Reduced Oxidative Stress Through Nrf‐2pathway In Vitro

**FIGURE 6:**
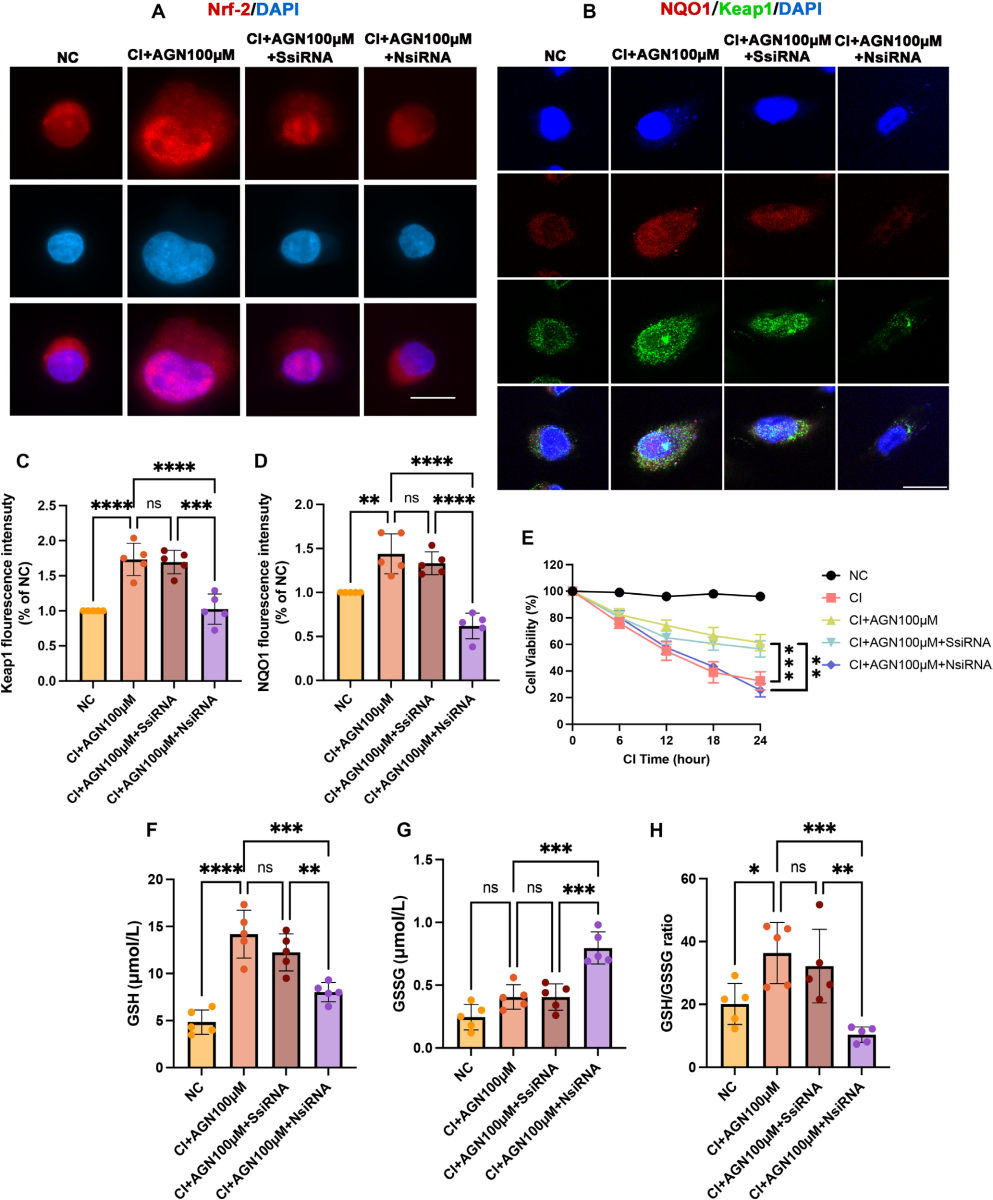
Agrimoniin activated the Nrf‐2 pathway by promoting Nrf‐2 translocation in QSG 7701 cells. The QSG 7701 cells were cold preserved in the UW preservation solution saturated with 100 μM agrimonii and/or the dissolved (200 μg in 10 mL of PBS) Nrf‐2 siRNA or scrambled siRNA at 4°C for 24 h. (A) Nrf‐2 (red) staining of the QSG 7701 cells. (B) NQO1 (red) and Keap1 (green) staining of the QSG 7701 cells. (C) Keap1 fluorescence intensity. (D) NQO1 fluorescence intensity. (I) Cell viability was evaluated by MTT assay. The concentrations of (F) GSH and (G) GSSG were evaluated and the (H) GSH/GSSG ratio was calculated. Nuclei were counterstained with DAPI (blue). Data were analysed using ANOVA with post hoc Bonferroni test and are presented as bars with scatter plots, mean ± SD. *n* = 5. Scale bar = 200 μm. *p < 0.05, **p < 0.01, ***p < 0.001, ****p < 0.0001.

To further confirm the role of Nrf‐2 in mediating the antioxidant effects of agrimoniin, immunofluorescence staining of Nrf‐2, NQO1, and Keap1 was performed in QSG 7701 cells and HepG2 cells subjected to CI. Agrimoniin supplementation markedly promoted nuclear translocation of Nrf‐2 (Figure 6A, Figure S1A) and increased the expression of its downstream effectors, NQO1 and Keap1 (*p* < 0.0001 and *p* < 0.01, Figure 6B–D, Figure S1B–D). However, these effects were significantly suppressed upon Nrf‐2 silencing, as shown by reduced nuclear Nrf‐2 accumulation, decreased NQO1 and Keap1 (*p* < 0.0001, Figure 6A–D, Figure S1A–D). Cell viability analysis revealed that agrimoniin significantly protected QSG 7701 cells from CI‐induced injury across various time points (*p* < 0.01–0.001, Figure 6E). In contrast, this protective effect was abolished in the presence of NsiRNA, with cell viability declining in a manner comparable to the CI group. Agrimoniin significantly increased GSH levels (*p* < 0.001), resulting in an elevated GSH/GSSG ratio (Figure 6F–H, Figure S1E–G). These oxidative protective effects were largely reversed by Nrf‐2 knockdown, as evidenced by the reduced GSH level (*p* < 0.05), elevated GSSG level (*p* < 0.001), and reduced GSH/GSSG ratio (*p* < 0.05, Figure 6E,F, Figure S1E–G).

### 3.7 Nrf‐2 Suppression Attenuated Agrimoniin‐Mediated Ferroptosis Reduction in In Vitro

**FIGURE 7:**
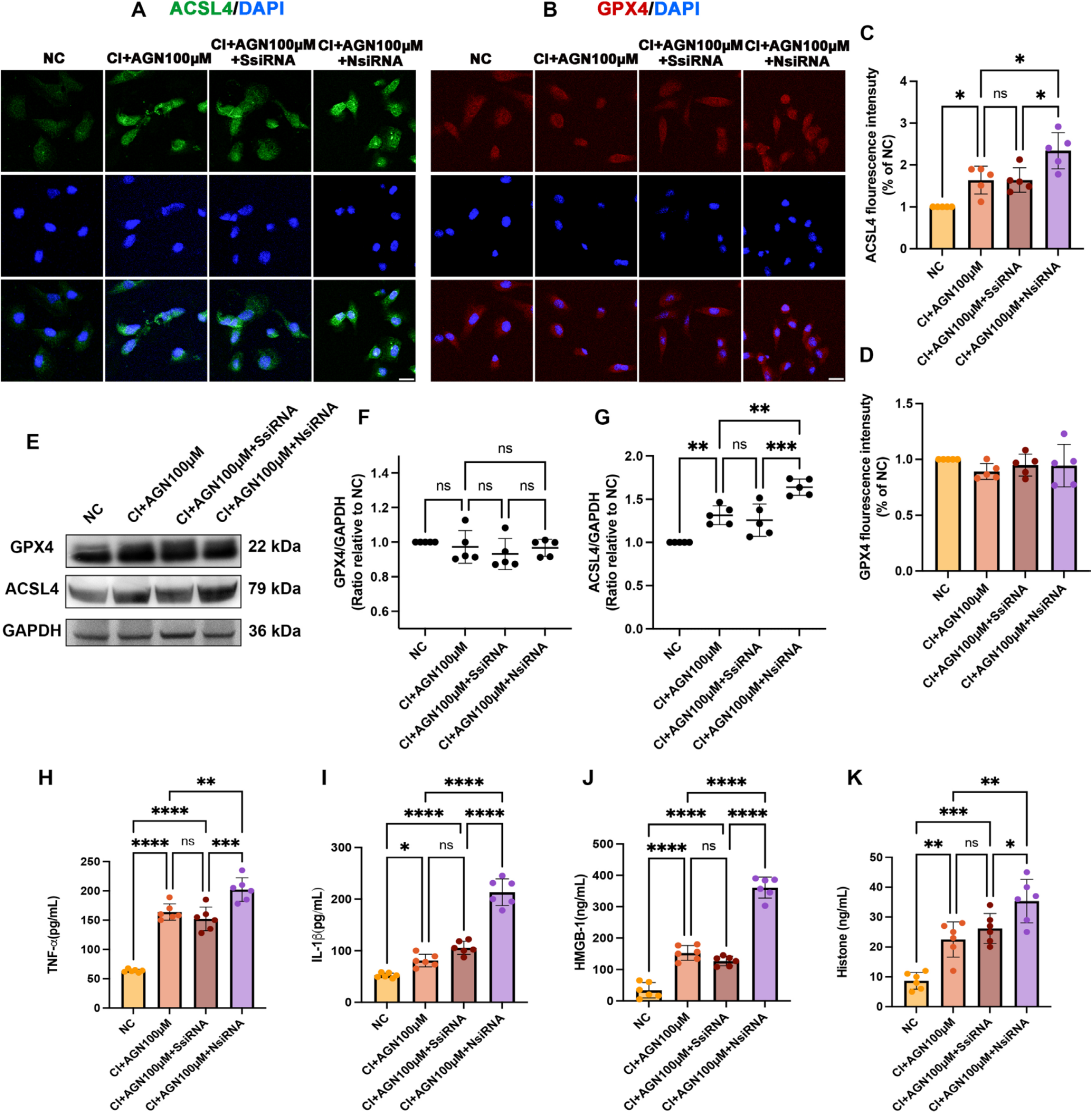
Nrf‐2 suppression attenuated agrimoniin‐mediated ferroptosis reduction in QSG 7701 cells. The QSG 7701 cells were cold preserved in the UW preservation solution saturated with 100 μM agrimonii and/or the dissolved (200 μg in 10 mL of PBS) Nrf‐2 siRNA or scrambled siRNA at 4°C for 24 h. (A) ACSL4 (green) staining of the QSG 7701 cells. (B) GPX4 (red) staining of the QSG 7701 cells. (C) ACSL4 fluorescence intensity. (D) GPX4 fluorescence intensity. (E) Ferroptosis was evaluated by detecting GPX4 and ACSL4 using Western Blot. Analysis shows the expression of (F) GPX4 and (G) ACSL4. The concentration of (H) TNF‐α, (I) IL‐1β, (J) HMGB‐1 and (K) Histone were evaluated by ELISA. Nuclei were counterstained with DAPI (blue). Data were analysed using ANOVA with post hoc Bonferroni test and are presented as bars with scatter plots, mean ± SD. *n* = 5. Scale bar = 200 μm. *p < 0.05, **p < 0.01, ***p < 0.001, ****p < 0.0001.

In CI QSG 7701 cells and HepG2 cells, Nrf‐2 silencing significantly enhanced the ferroptosis evidenced by increased ACSL4 expression (*p* < 0.05, Figure 7A,C, Figure S2A), but GPX4 expression showed no significant change (*p* > 0.05, Figure 7B,D, Figure S2C). In the meantime, the western blot showed consistent results that Nrf‐2 knockdown significantly increased ACSL4 expression compared to the agrimoniin supplemented group (*p* < 0.001, Figure 7E,G, Figure S2D,F), while GPX4 expression showed no significant difference across all groups (Figure 7F, Figure S2E). In addition, the anti‐inflammatory effects of agrimoniin were significantly abolished following Nrf‐2 silencing in QSG 7701 cells and HepG2 cells, with the levels of TNF‐α, IL‐1β, HMGB1, and histone markedly increased compared to the agrimoniin treated group (*p* < 0.05 to 0.0001, Figure 7H–K, Figure S2G–J).

## 4 Discussion

For the first time, the present study demonstrated that supplementation of a high dose of agrimoniin (100 μM) into the UW cold storage solution significantly protected liver grafts from DCD rats by reducing liver architecture damage, ferroptosis, and pro‐inflammatory cytokines and DAMPs release. This protective effect of agrimoniin may be mediated by its antioxidative property via activating the Nrf‐2 pathway. Meanwhile, intraperitoneally injected agrimoniin also reduced cardiac death and warm ischemia‐induced liver morphology damage and ferroptosis, further demonstrating the anti‐ischemia effect of agrimoniin (summarised in Graphical Abstract). In vitro, agrimoniin protected human normal liver cells, QSG 7701 cell from cold preservation induced ferroptosis and oxidative stress, and activated the Nrf‐2 pathway by promoting Nrf‐2 translocation. Collectively, those data demonstrated the therapeutic potential of agrimoniin on improving DVD liver grafts quality through its anti‐ferroptosis and anti‐oxidative stress effect.

IR frequently occurs as an injury during liver transplantation [14, 15]. Typical features of hepatic IR injury include iron overload and lipid peroxidation [16]. Recent research has shown that IR can induce ferroptosis in hepatocytes due to the overproduction of reactive oxygen species (ROS) and overload iron, as the liver is susceptible to oxidative damage [17]. In the context of liver transplantation, IR injury may contribute to primary graft dysfunction (PGD), delayed graft function (DGF) or acute rejection, resulting in poorer recipient outcomes such as increased rates of graft loss and mortality [18, 19]. Therefore, therapeutic approaches to IR injury can effectively improve liver graft quality and improve transplantation prognosis.

In this study, we observed a notable increase in the expression of the ferroptosis biomarker ACSL4 in DCD livers. Furthermore, subjecting DCD livers to 24 h of cold storage exacerbated ferroptosis. These findings suggested that using liver grafts from DCD donors may elevate the risk of post‐transplantation complications. Our results also suggested liver protection against ferroptosis with the supplementation of agrimoniin into the storage solution. However, the expression of GPX4, a key inhibitor of ferroptosis through its lipid peroxide–reducing activity [20, 21], was not altered by agrimoniin, suggesting that alternative inhibitory pathways may be involved. Ferroptosis can be suppressed through several GPX4‐independent mechanisms, including the FSP1–CoQ10 axis [22], the GCH1–BH4 pathway [23], and iron‐metabolism related proteins such as FTH1 and FPN1 [24]. Since ferroptosis is primarily triggered by lipid peroxidation, and agrimoniin exhibits strong antioxidative properties and potent radical‐scavenging activity, it is likely that agrimoniin inhibits lipid peroxidation upstream of GPX4 or activates parallel antioxidant systems without altering GPX4 protein abundance. Our findings propose agrimoniin as a potential therapeutic approach to enhance the quality of DCD liver grafts for improved clinical outcomes, although further investigations are warranted.

Agrimoniin, classified as a hydrolysable tannin, is a polyphenolic compound known for its antioxidant and anti‐inflammatory characteristics [25, 26]. Agrimoniin exhibits potent antioxidant activity, which helps scavenge free radicals and prevent oxidative stress‐related damage within the body [27]. Earlier investigations have revealed the efficacy of agrimoniin in scavenging various free radicals, encompassing DPPH radicals and superoxide anions, indicating its strong antioxidant potential [28, 29]. The anti‐inflammatory activity of agrimoniin in macrophages has been reported due to its NO scavenging ability and attenuation of the expression of inflammatory markers such as TNF‐α, IL‐6, and cyclooxygenase‐2 (COX‐2) [25, 30]. Our findings in this study further demonstrated that agrimoniin could protect prolonged cold‐preserved DCD liver grafts through these protective properties.

The Nrf2 pathway plays a central role in regulating the expression of antioxidant and detoxification genes to maintain cellular homeostasis [31, 32]. Several downstream effectors of Nrf2 are implicated in ferroptosis regulation. These include SLC7A11, which promotes cystine import and GSH synthesis [33]; HO‐1 and FTH1, which modulate intracellular iron pools [34]. NQO1 is one of the key downstream targets of Nrf2. It catalyses the reduction of quinones and quinone derivatives, thereby preventing the generation of ROS and protecting cells from oxidative damage [35]. Keap1 is the negative regulator of Nrf2, acting as a substrate adaptor for the Cullin 3 (Cul3)‐dependent ubiquitin ligase complex, facilitating the ubiquitination and proteasomal degradation of Nrf2 [36]. Nrf2 activation upregulates the expression of antioxidant enzymes such as superoxide dismutase (SOD), catalase, and glutathione peroxidase, which scavenge ROS and reduce oxidative damage and following inflammation responses [37]. Our study demonstrated that the agrimoniin treatment significantly upregulated Nrf2 and its downstream effector NQO1, supporting the notion that activation of the Nrf2–NQO1 axis plays a pivotal role in mediating its protective effects. Given the role of NQO1 in maintaining redox homeostasis, this pathway likely contributes substantially to the preservation of DCD liver grafts by suppressing ferroptotic injury. Taken together, agrimoniin targeting the Nrf2 pathway represented a promising therapeutic strategy for mitigating DCD liver graft damage before transplantation.

In clinical organ preservation solutions, various antioxidant or cytoprotective additives are included to minimise IR injury. In the UW solution used in our study, GSH and allopurinol are incorporated as key antioxidant components [38]. GSH functions as a reducing agent within the GSH/GSSG cycle to counteract oxidative damage [39], which partially overlaps with the antioxidative mechanism of agrimoniin. Notably, despite the presence of exogenous GSH in the preservation solution, agrimoniin further enhanced endogenous GSH level, highlighting its potent capability to reinforce endogenous antioxidant defences. In addition to the antioxidant mentioned above, other additives such as mannitol and tryptophan are used in HTK solution, maintaining cell integrity by preventing osmotic swelling and stabilising cellular membranes [40]. These mechanisms differ from that of agrimoniin, suggesting potential complementarity. Future studies could investigate whether combining agrimoniin with existing preservation additives can further enhance organ protection.

In this study, supplementing agrimoniin into the cold storage solution effectively mitigated ischemic injury in DCD liver grafts, suggesting potential value in improving graft quality and ultimately enhancing recipient outcomes. Although agrimoniin has been reported to exert antioxidant, anti‐inflammatory, and mitochondrial‐modulating effects, existing evidence—including our findings—remains predominantly preclinical [26, 41]. Further clarification of its molecular targets, pharmacokinetics, and standardised formulation will be essential to fully establish its safety and efficacy before agrimoniin can advance towards clinical application.

## Author Contributions

Conceptualisation: Enqiang Chang and Jiaqiang Zhang. Methodology: Enqiang Chang and Xiaoguo Ruan. Investigation: Xiaoguo Ruan, Ningtao Li, Yongfeng Zhu, Hongyan Miu, Yunting Liu and Enqiang Chang. Visualisation: Xiaoguo Ruan, Ningtao Li and Enqiang Chang. Supervision: Jiaqiang Zhang. Writing – original draft: Xiaoguo Ruan and Ningtao Li. Writing – review and editing: Enqiang Chang and Jiaqiang Zhang. All data was generated in‐house, and no paper mill was used. All authors agree to be accountable for all aspects of work ensuring integrity and accuracy.

## Funding

This work was supported by the Natural Science Foundation of Henan Province (314 242300421192, JQRC2023004).

## Conflicts of Interest

The authors declare no conflicts of interest.

## Supporting information


**Figure S1:** Agrimoniin activated Nrf‐2 pathway by promoting Nrf‐2 translocation in HepG2 cells.
The HepG2 cells were cold preserved in the UW preservation solution saturated with 100 μM agrimoniin and/or the dissolved (200 μg in 10 mL of PBS) Nrf‐2 siRNA or scrambled siRNA at 4°C for 24 h. (A) Nrf‐2 (red) staining of the HepG2 cells. (B) NQO1 (red) and Keap1 (green) staining of the HepG2 cells. (C) Keap1 fluorescence intensity. (D) NQO1 fluorescence intensity. (I) Cell viability was evaluated by MTT assay. The concentration of (F) GSH and (G) GSSG were evaluated and the (H) GSH/GSSG ratio was calculated. Nuclei were counterstained with DAPI (blue). Data were analysed using ANOVA with post hoc Bonferroni test and are presented as bars with scatter plots, mean ± SD. *n* = 5. Scale bar = 200 μm. *p < 0.05, **p < 0.01, ***p < 0.001, ****p < 0.0001.
**Figure S2:** Nrf‐2 suppression attenuated agrimoniin‐mediated ferroptosis reduction in HepG2 cells.
The HepG2 cells were cold preserved in the UW preservation solution saturated with 100 μM agrimoniin and/or the dissolved (200 μg in 10 mL of PBS) Nrf‐2 siRNA or scrambled siRNA at 4°C for 24 h. (A) ACSL4 (green) staining of the QSG 7701 cells. (B) GPX4 (red) staining of the HepG2 cells. (C) ACSL4 fluorescence intensity. (D) GPX4 fluorescence intensity. (E) Ferroptosis was evaluated by detecting GPX4 and ACSL4 using Western Blot. Analysis shows the expression of (F) GPX4 and (G) ACSL4. The concentration of (H) TNF‐α, (I) IL‐1β, (J) HMGB‐1 and (K) Histone were evaluated by ELISA. Nuclei were counterstained with DAPI (blue). Data were analysed using ANOVA with post hoc Bonferroni test and are presented as bars with scatter plots, mean ± SD. *n* = 5. Scale bar = 200 μm. *p < 0.05, **p < 0.01, ***p < 0.001, ****p < 0.0001.

**Data S1:** Supporting Information.

## Data Availability

The data that support the findings of this study are available on request from the corresponding author. The data are not publicly available due to privacy or ethical restrictions.
