# Supplementary material for: Agrimoniin Alleviates Ferroptosis in Cold‐Stored DCD Liver Grafts Through Activation of the Nrf‐2 Pathway
Source: Cell Prolif. 2026 Jan 20;59(7):e70164. doi: 10.1111/cpr.70164 (PMC13325472; doi:10.1111/cpr.70164)
Supplement: Supplementary file 1 — Figure S1: Agrimoniin activated Nrf‐2 pathway by promoting Nrf‐2 translocation in HepG2 cells. The HepG2 cells were cold preserved in the UW preservation solution saturated with 100 μM agrimoniin and/or the dissolved (200 μg in 10 mL of PBS) Nrf‐2 siRNA or scrambled siRNA at 4°C for 24 h. (A) Nrf‐2 (red) staining of the HepG2 cells. (B) NQO1 (red) and Keap1 (green) staining of the HepG2 cells. (C) Keap1 fluorescence intensity. (D) NQO1 fluorescence intensity. (I) Cell viability was evaluated by MTT assay. The concentration of (F) GSH and (G) GSSG were evaluated and the (H) GSH/GSSG ratio was calculated. Nuclei were counterstained with DAPI (blue). Data were analysed using ANOVA with post hoc Bonferroni test and are presented as bars with scatter plots, mean ± SD. n = 5. Scale bar = 200 μm. *p < 0.05, **p < 0.01, ***p < 0.001, ****p < 0.0001. Figure S2: Nrf‐2 suppression attenuated agrimoniin‐mediated ferroptosis reduction in HepG2 cells. The HepG2 cells were cold preserved in the UW preservation solution saturated with 100 μM agrimoniin and/or the dissolved (200 μg in 10 mL of PBS) Nrf‐2 siRNA or scrambled siRNA at 4°C for 24 h. (A) ACSL4 (green) staining of the QSG 7701 cells. (B) GPX4 (red) staining of the HepG2 cells. (C) ACSL4 fluorescence intensity. (D) GPX4 fluorescence intensity. (E) Ferroptosis was evaluated by detecting GPX4 and ACSL4 using Western Blot. Analysis shows the expression of (F) GPX4 and (G) ACSL4. The concentration of (H) TNF‐α, (I) IL‐1β, (J) HMGB‐1 and (K) Histone were evaluated by ELISA. Nuclei were counterstained with DAPI (blue). Data were analysed using ANOVA with post hoc Bonferroni test and are presented as bars with scatter plots, mean ± SD. n = 5. Scale bar = 200 μm. *p < 0.05, **p < 0.01, ***p < 0.001, ****p < 0.0001. [file CPR-59-e70164-s002.docx]

**Figure S1:**


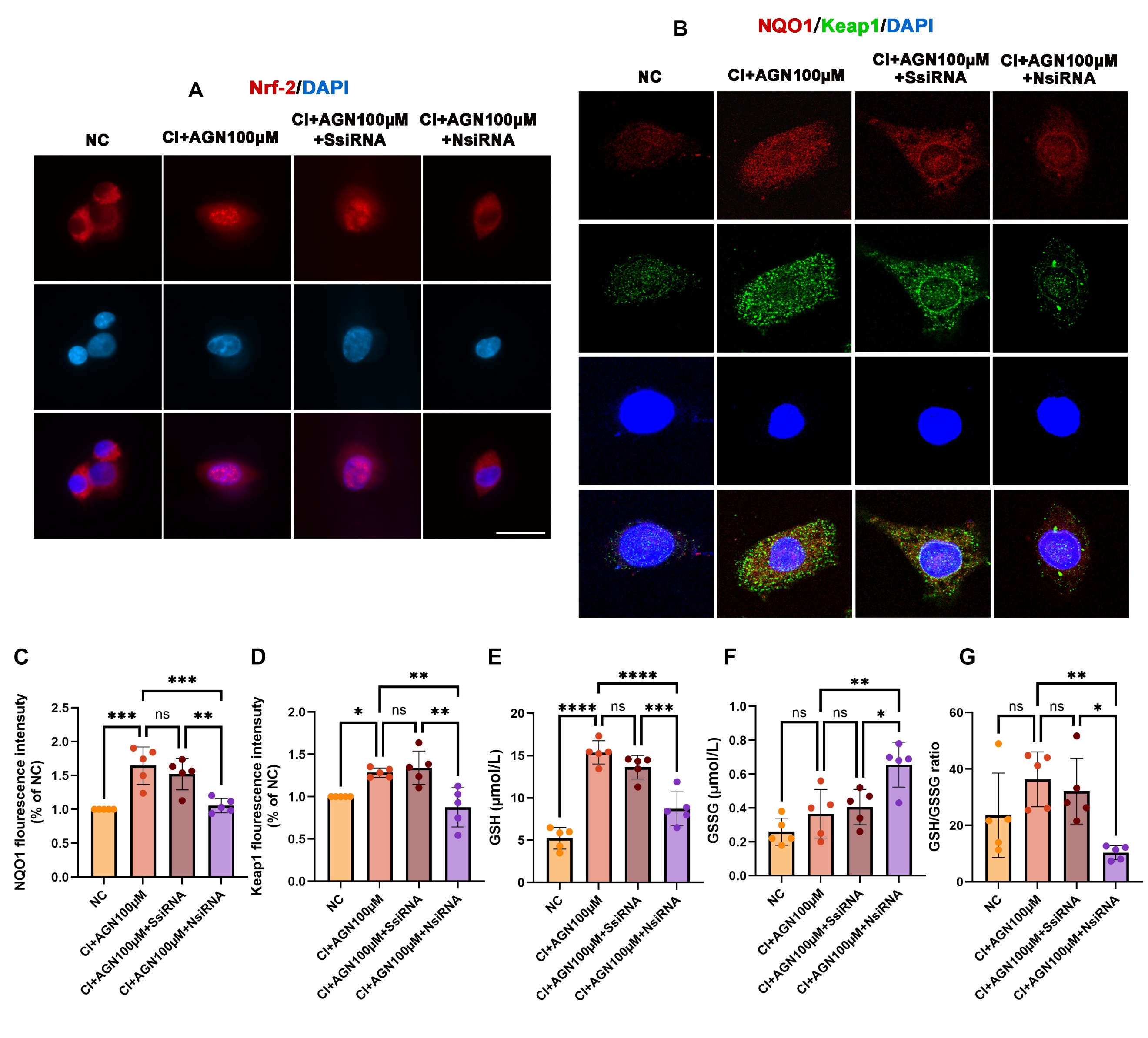


**S1. Agrimoniin activated Nrf-2 pathway by promoting Nrf-2 translocation in HepG2 cells**

The HepG2 cells were cold preserved in the UW preservation solution saturated with 100 µM agrimoniin and/or the dissolved (200 µg in 10 mL of PBS) Nrf-2 siRNA or scrambled siRNA at 4 ˚C for 24 h. (A) Nrf-2 (red) staining of the HepG2 cells. (B) NQO1 (red) and Keap1 (green) staining of the HepG2 cells. (C) Keap1 fluorescence intensity. (D) NQO1 fluorescence intensity. (I) Cell viability was evaluated by MTT assay. The concentration of (F) GSH and (G) GSSG were evaluated and the (H) GSH/GSSG ratio was calculated. Nuclei were counterstained with DAPI (blue). Data were analysed using ANOVA with post-hoc Bonferroni test and are presented as bars with scatter plots, mean ± SD. n = 5. Scale bar = 200 µm. *p < 0.05, **p < 0.01, ***p < 0.001, ****p < 0.0001.

**Figure S2:**

**
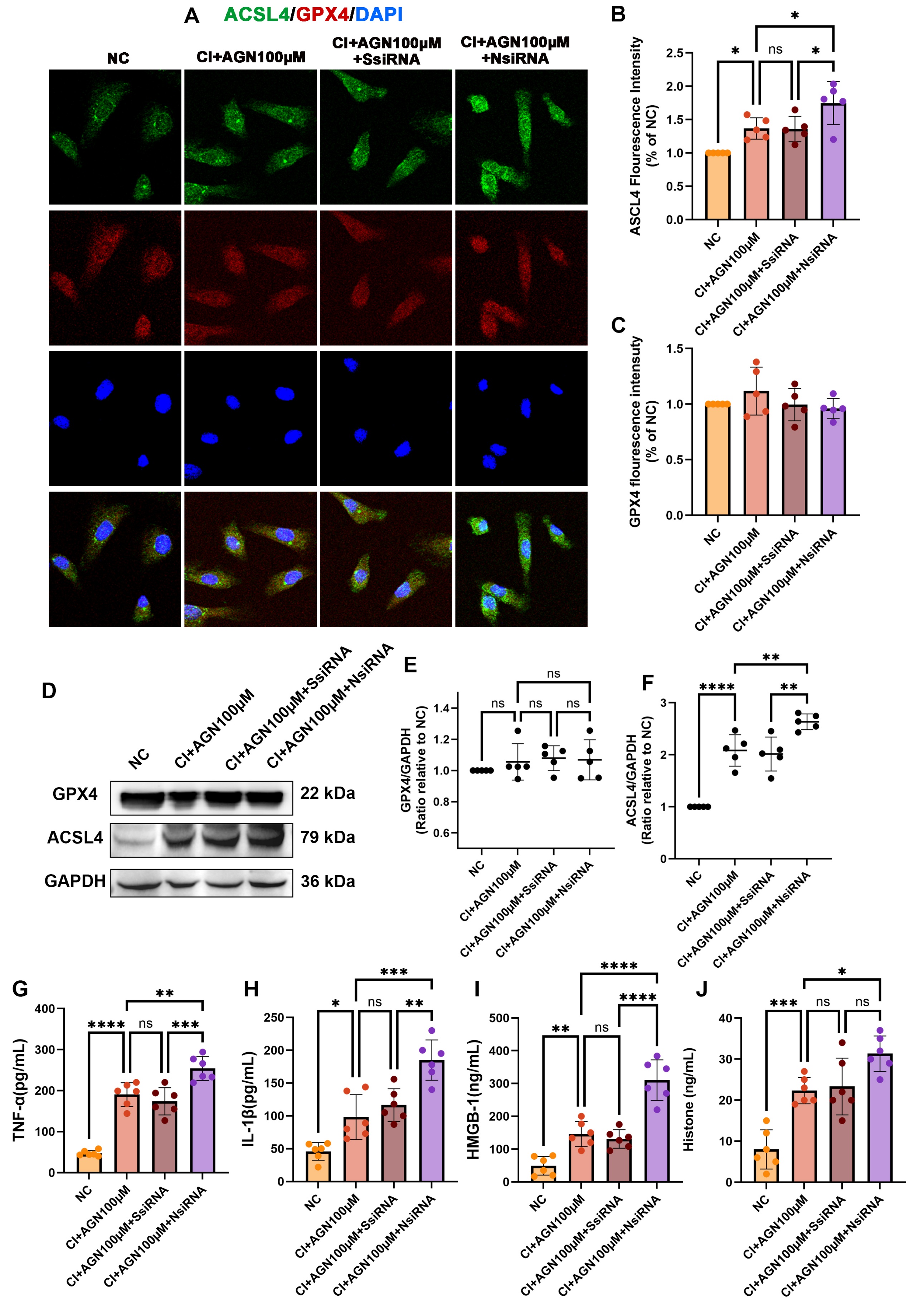
**

**S2. Nrf-2 suppression attenuated agrimoniin-mediated ferroptosis reduction in HepG2 cells.**

The HepG2 cells were cold preserved in the UW preservation solution saturated with 100 µM agrimoniin and/or the dissolved (200 µg in 10 mL of PBS) Nrf-2 siRNA or scrambled siRNA at 4 ˚C for 24 h. (A) ACSL4 (green) staining of the QSG 7701 cells. (B) GPX4 (red) staining of the HepG2 cells. (C) ACSL4 fluorescence intensity. (D) GPX4 fluorescence intensity. (E) Ferroptosis was evaluated by detecting GPX4 and ACSL4 using Western Blot. Analysis shows the expression of (F) GPX4 and (G) ACSL4. The concentration of (H) TNF-α, (I) IL-1β, (J) HMGB-1 and (K) Histone were evaluated by ELISA. Nuclei were counterstained with DAPI (blue). Data were analysed using ANOVA with post-hoc Bonferroni test and are presented as bars with scatter plots, mean ± SD. n = 5. Scale bar = 200 µm. *p < 0.05, **p < 0.01, ***p < 0.001, ****p < 0.0001.
