# Supplementary material for: Agrimoniin Alleviates Ferroptosis in Cold‐Stored DCD Liver Grafts Through Activation of the Nrf‐2 Pathway
Source: Cell Prolif. 2026 Jan 20;59(7):e70164. doi: 10.1111/cpr.70164 (PMC13325472; doi:10.1111/cpr.70164)

Full unedited blot and gel images 4I CHOP

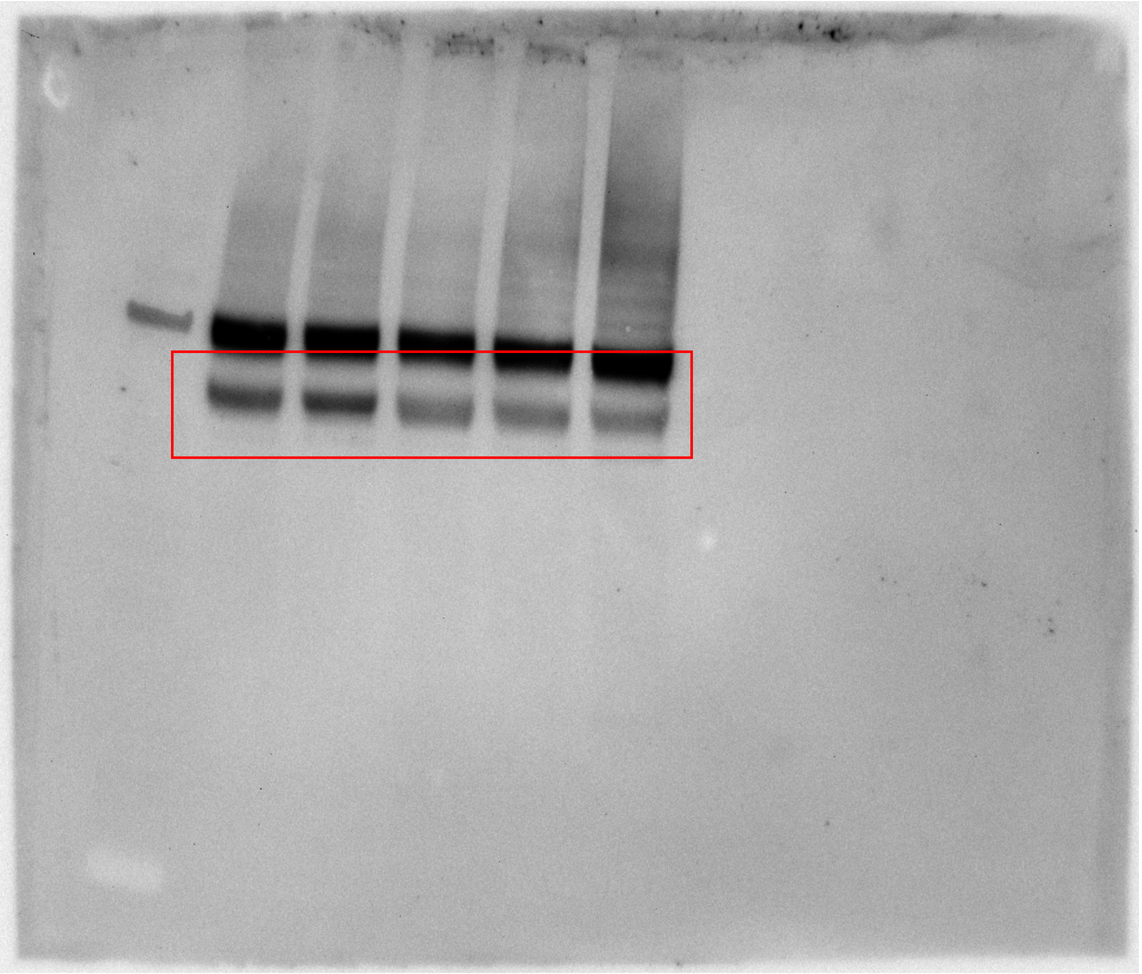

CHOP

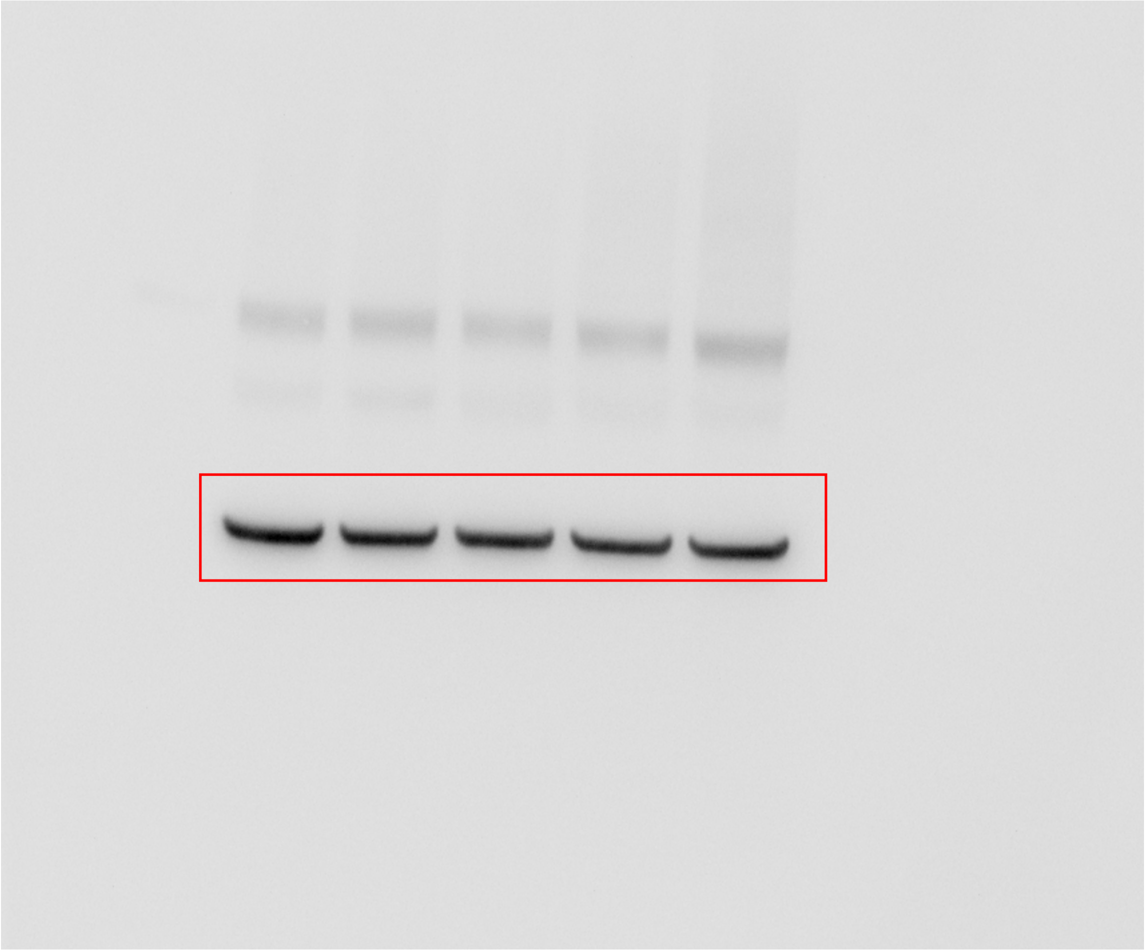

GAPDH

Full unedited blot and gel images 4I GRP78

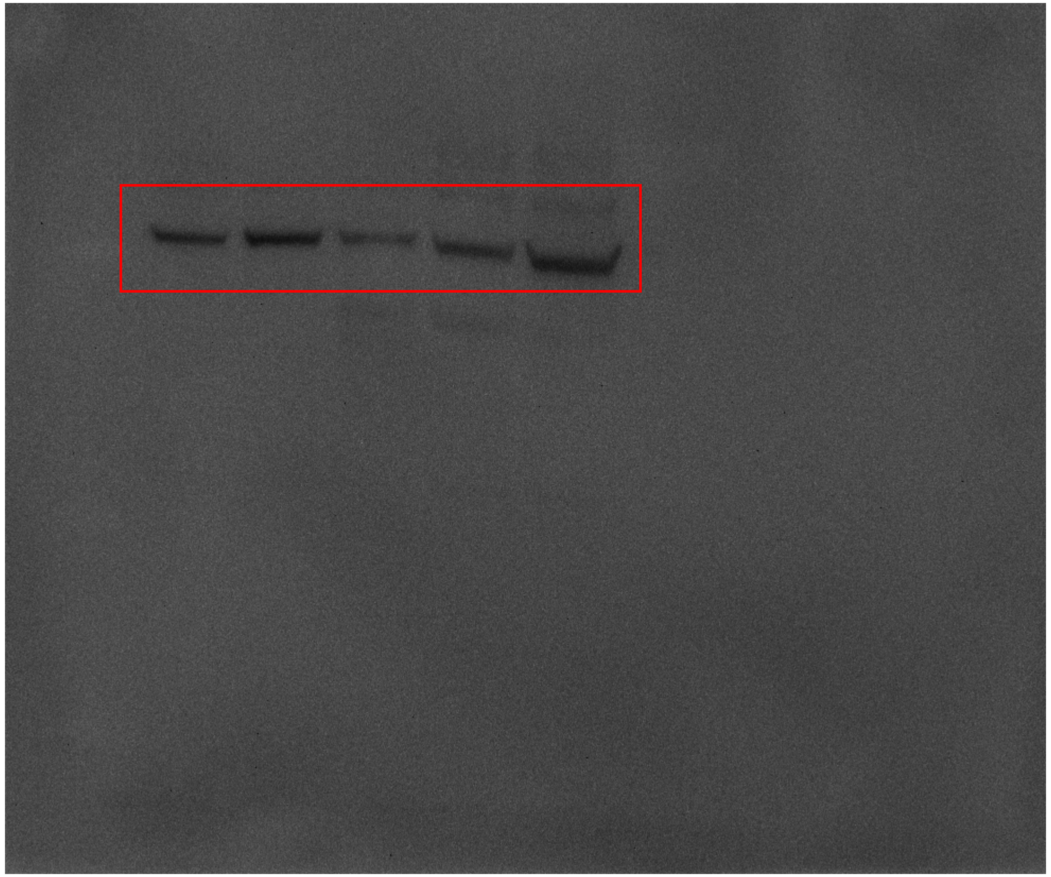

GRP78

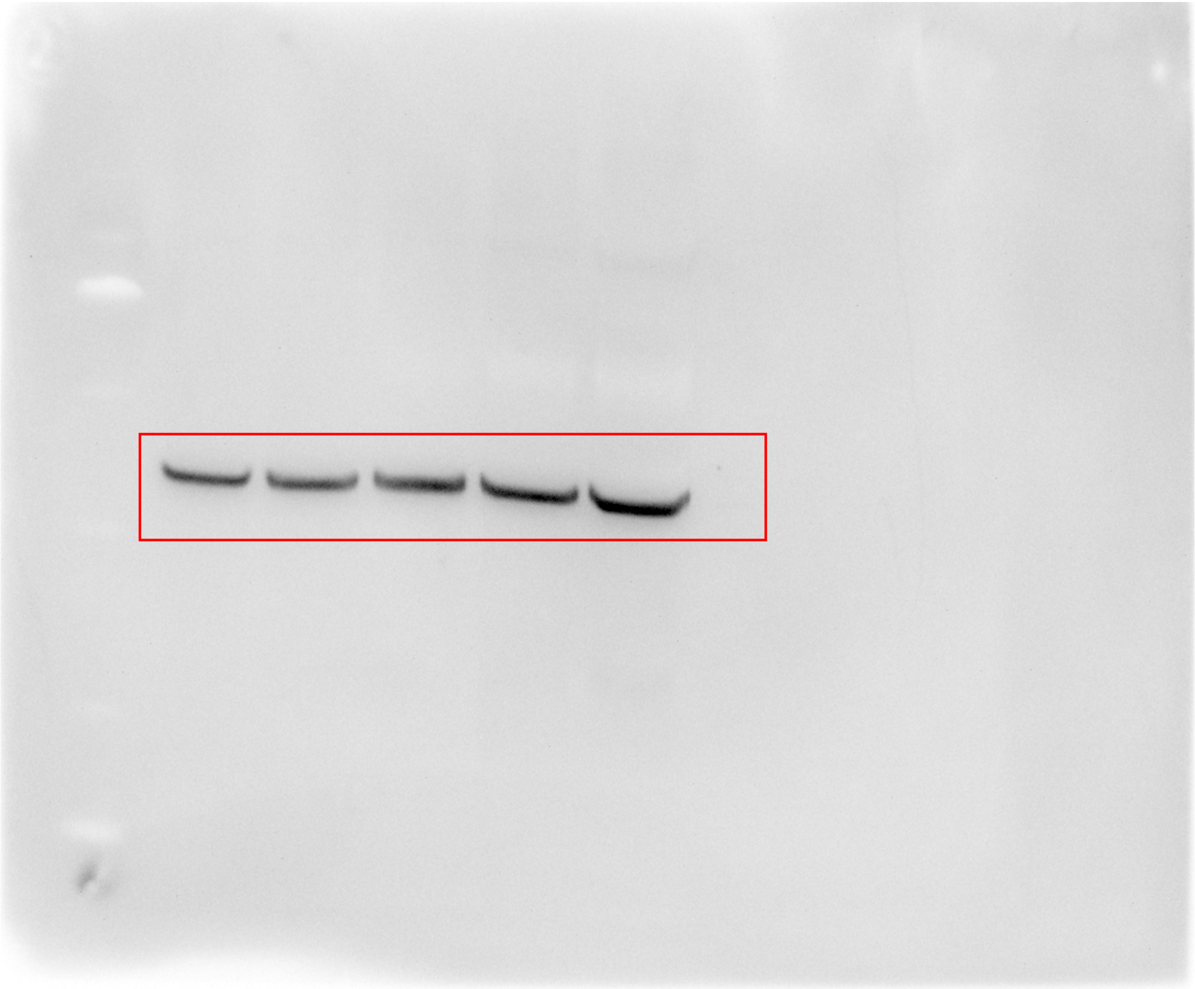

GAPDH

Full unedited blot and gel images 5I GPX4

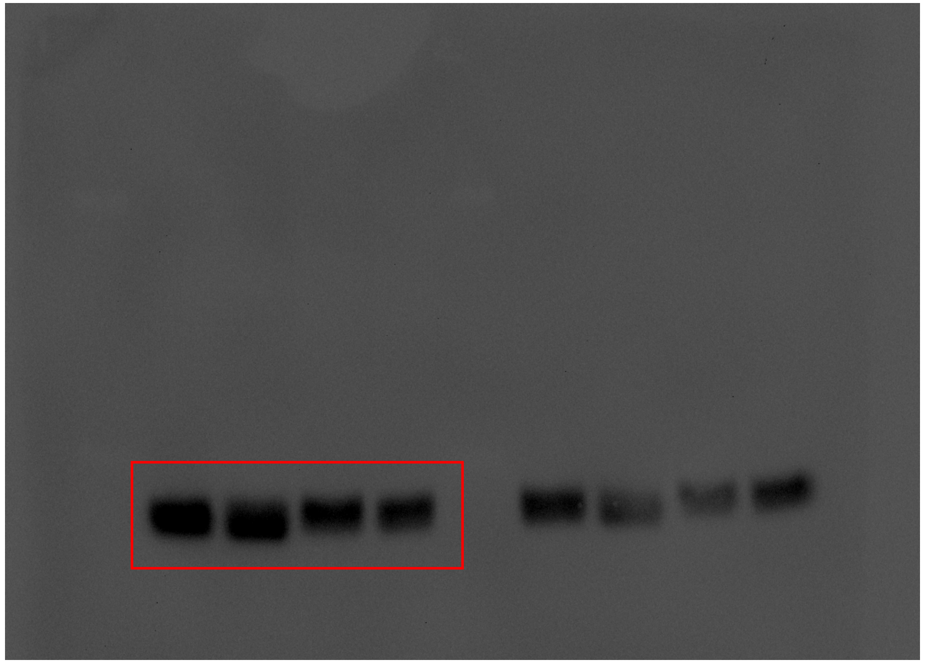

GPX4

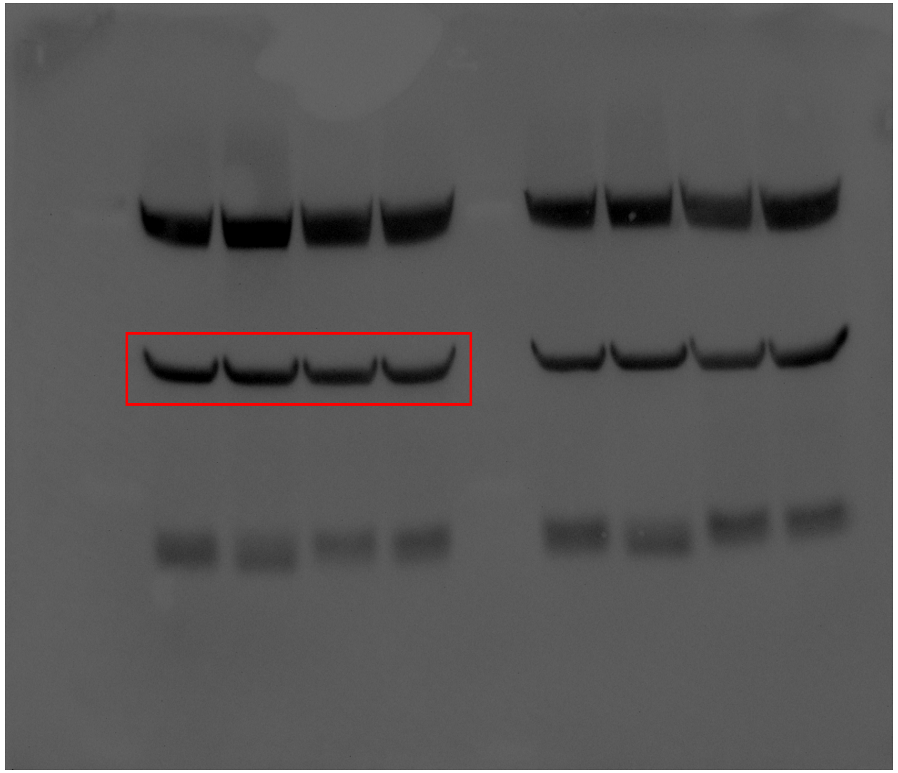

GAPDH

Full unedited blot and gel images 5I ACSL4

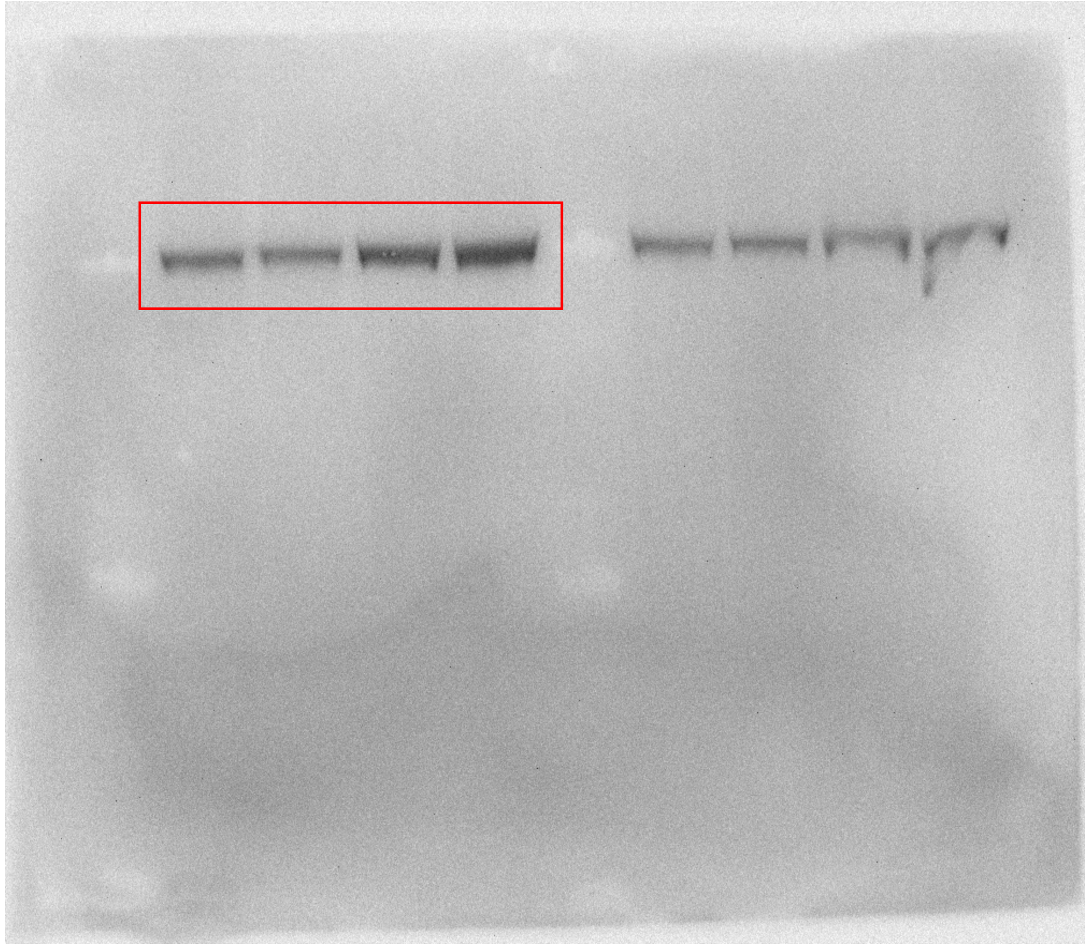

GRP78

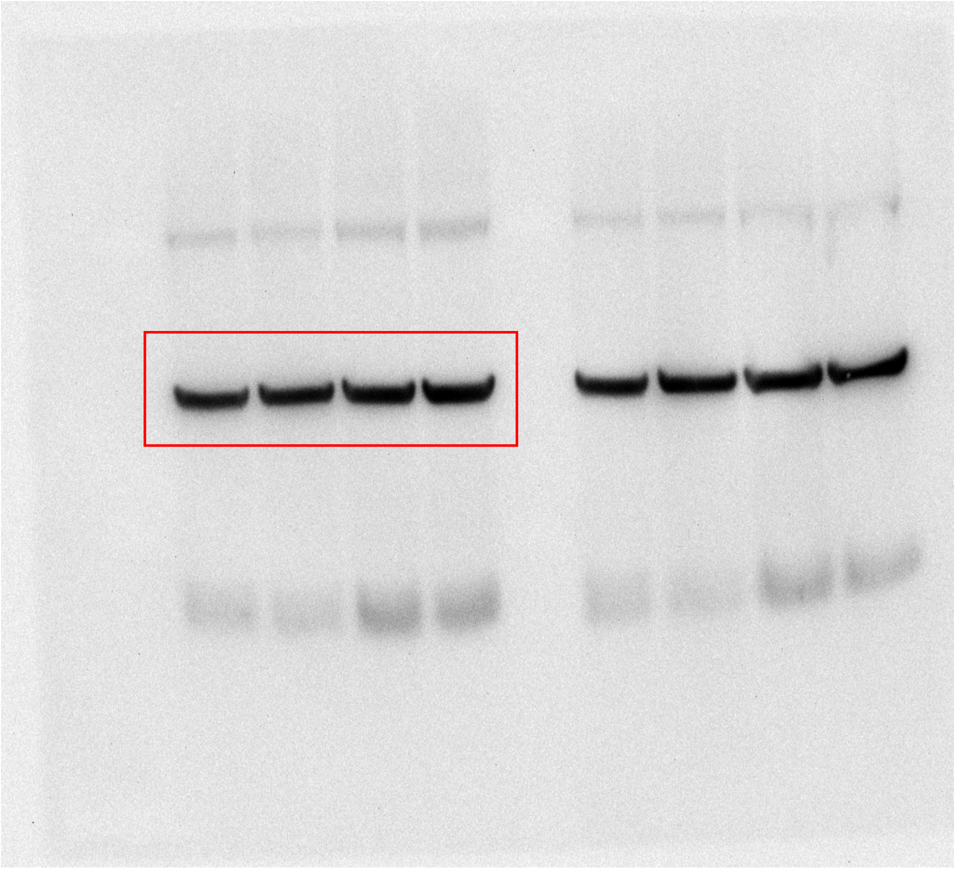

GAPDH

Full unedited blot and gel images 6E GPX4

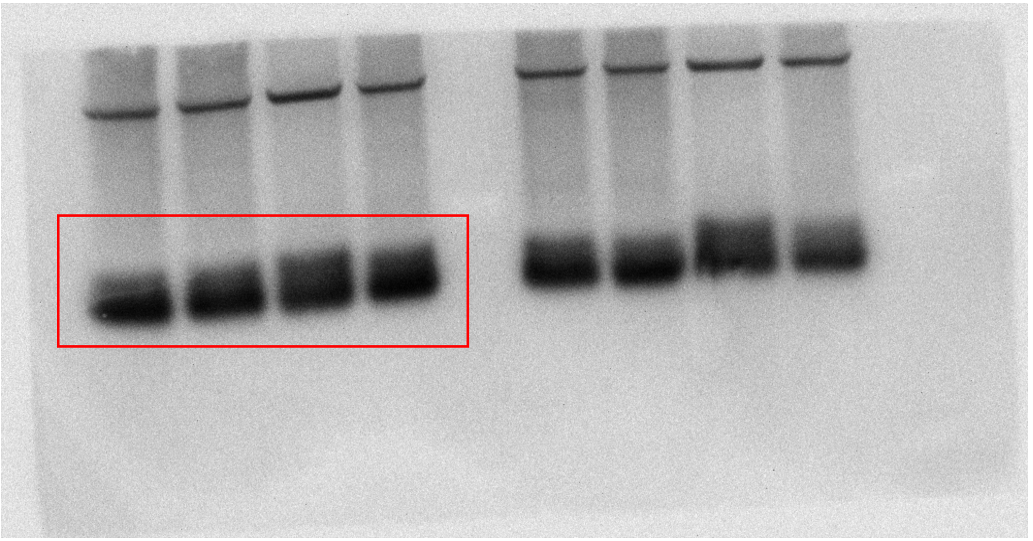

GPX4

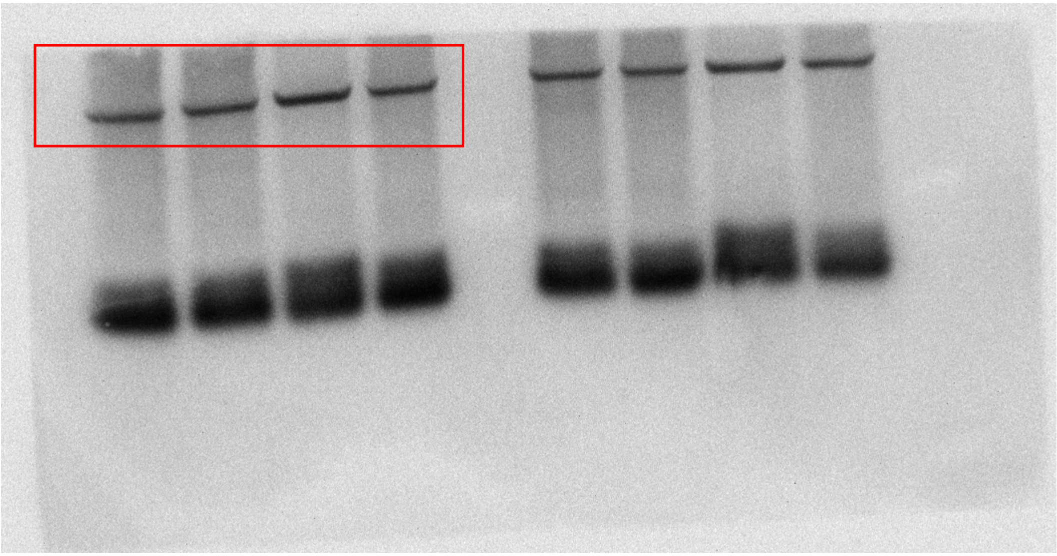

GAPDH

Full unedited blot and gel images 6E

ACSL4

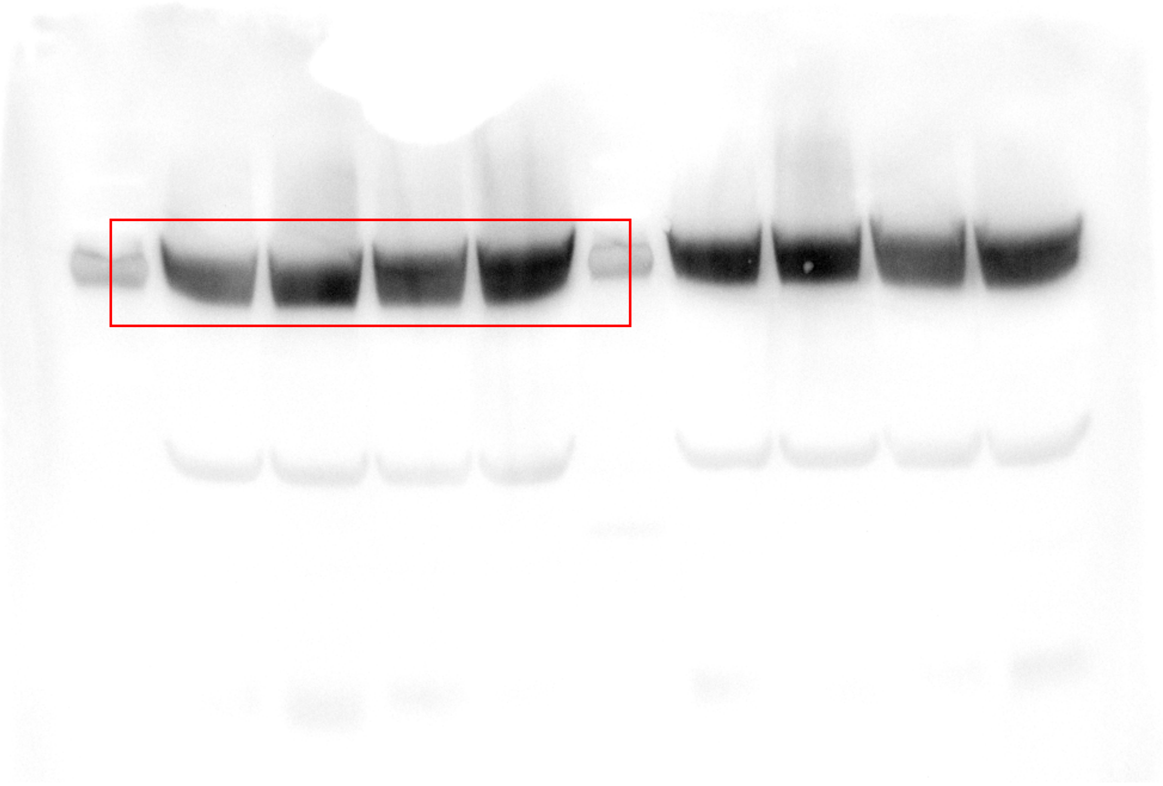

ACSL4

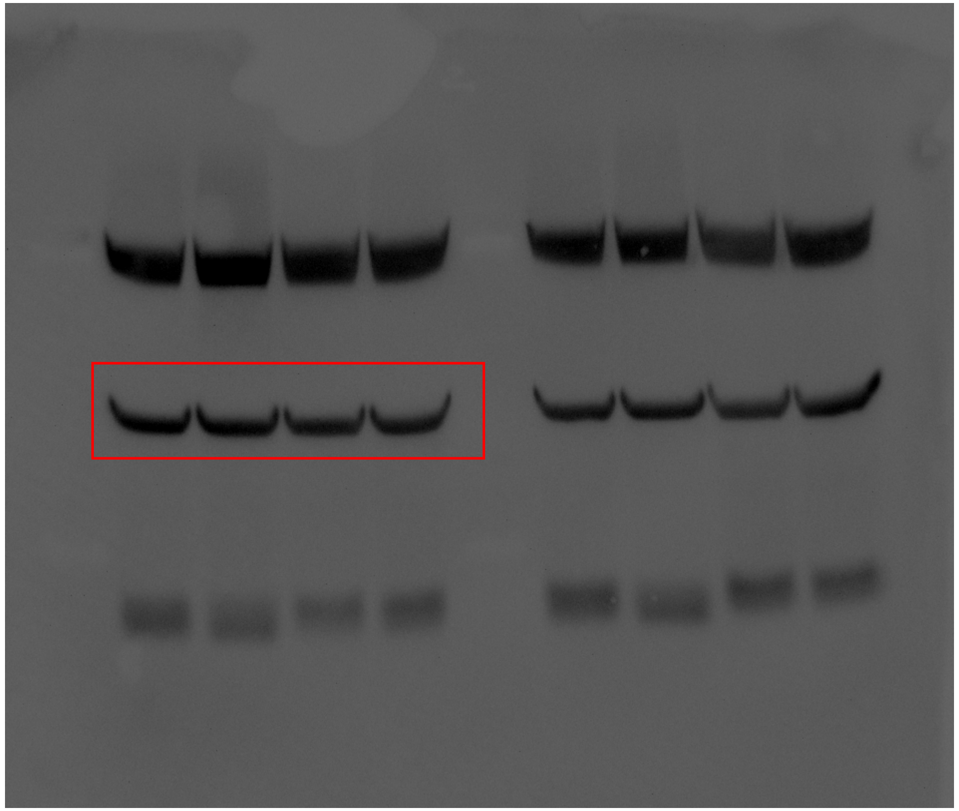

GAPDH

Full unedited blot and gel images S2

ACSL4

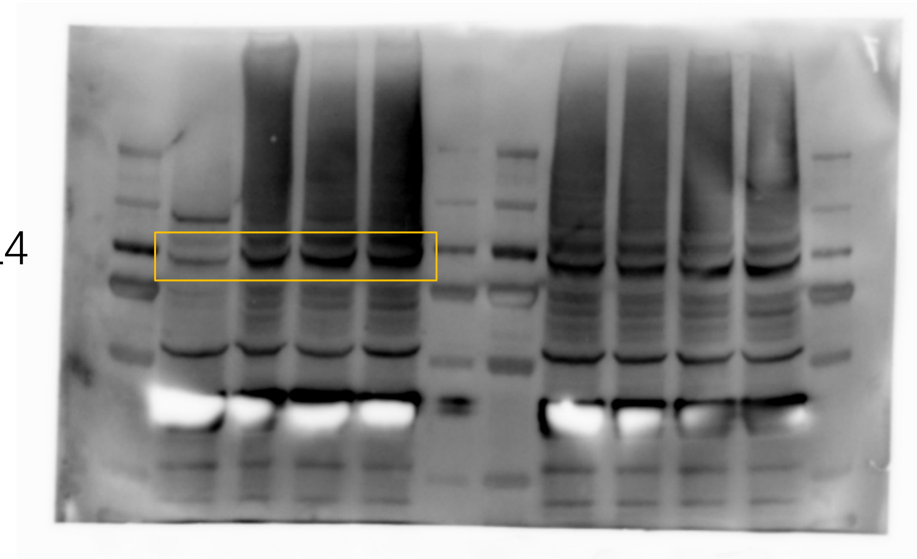

GAPDH

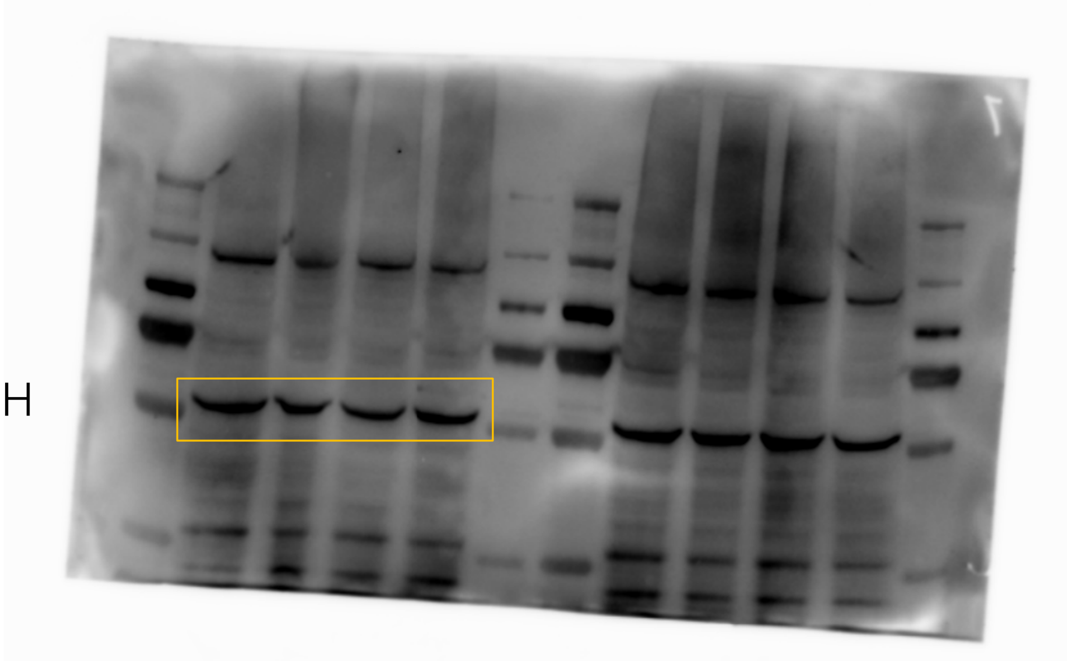

GPX4

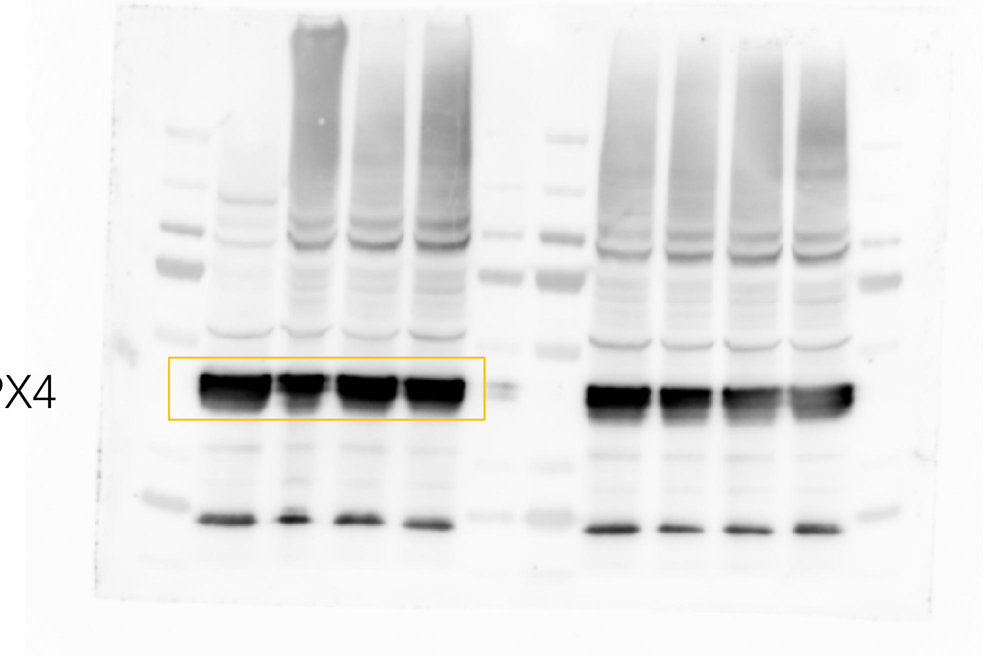

Supplement: Supplementary file 2 — Data S1: Supporting Information. [file CPR-59-e70164-s001.pdf]
